# Causal impact of elevated body mass index on diabetic kidney disease: an integrated Mendelian randomization and Global Burden of Disease Study 2021 analysis

**DOI:** 10.1080/0886022X.2025.2472981

**Published:** 2025-03-17

**Authors:** Ye-xin Chen, Dong-sen Hu, Mao-xuan Lin, Zi-heng Gao, Han-zhang Hong, Yu-xin Hu, Ling-zi Yao, Gai-wen Cui, Lin Wang

**Affiliations:** a Dongzhimen Hospital, Beijing University of Chinese Medicine, Beijing, China; b Beijing University of Chinese Medicine, Beijing, China; c School of Public Health, Zhejiang University School of Medicine, Hangzhou, China

**Keywords:** Mendelian Randomization, Global Burden of Disease Study 2021, diabetic kidney disease, elevated body mass index, type 2 diabetes mellitus, chronic kidney disease

## Abstract

**Background:**

Elevated body mass index (BMI) has been implicated in the pathogenesis of diabetic kidney disease among patients with type 2 diabetes mellitus (T2DKD). However, establishing a causal relationship and quantifying the resultant global health impact remain challenging.

**Methods:**

A two-sample Mendelian randomization (MR) analysis was conducted using summary-level data obtained from the IEU database. Multiple MR approaches, including inverse variance weighted (IVW), MR-Egger regression, weighted median, weighted mode, and simple mode methods, were implemented to ensure robust causal inference. In parallel, Global Burden of Disease Study (GBD) 2021 were analyzed to determine the trends in mortality and disability-adjusted life years (DALYs) in T2DKD attributable to high BMI (HBMI-T2DKD) from 1990 to 2021. Joinpoint regression was used to estimate the average annual percent change (AAPC). Bayesian age-period-cohort (BAPC) models were then applied to project the disease burden through 2049.

**Results:**

MR analyses provided strong evidence for a causal relationship between elevated BMI and T2DKD. The GBD analysis revealed a sustained global increase in HBMI-T2DKD burden over the past three decades. Between 1990 and 2021, the result of AAPC indicated a persistent upward trend. The burden was particularly high among older adults, with the highest impact observed in East Asia and middle Socio-Demographic Index (SDI) region. By 2049, HBMI-T2DKD-related disease burden were projected to continue rising.

**Conclusions:**

Elevated BMI is a significant causal risk factor for T2DKD. The integration of MR and GBD 2021 data underscores the urgent need for targeted public health interventions to reduce BMI levels, especially in high-risk regions and aging populations.

## 1. Introduction

Diabetic kidney disease (DKD) is a prevalent chronic consequence of type 2 diabetes mellitus (T2DM), affecting around 20% to 40% of T2DM patients [1,2]. From 1990 to 2017, the global new cases of chronic kidney disease due to diabetes mellitus type 2 (T2DKD) increased from 1.4 million to 2.4 million, representing a roughly 74% rise [3], which resulted in a substantial economic and social burden on a global scale [4,5]. In the medicare population alone, DKD-related expenditures were nearly $25 billion in 2011 [6]. Over the years, this has increasingly imposed heavy medical and economic burdens globally [7].

Multiple studies have shown that hypertension, hyperglycemia, hyperlipidemia may accelerate the development and advancement of T2DKD. Out of these factors, high body mass index (HBMI) is an important risk factor that should not be ignored. An extensive meta-analysis involving 41,271 patients with T2DM demonstrated that there was a 16% increase in the probability of developing T2DKD for every 5 kg/m^2^ increase in BMI [8]. A prospective cohort study observed that an elevation in BMI significantly heightened the incidence of T2DKD in a sample of 2,959 patients with T2DM [9]. These studies emphasized the importance of BMI in the development of T2DKD. Additionally, a retrospective cohort study confirmed a significant correlation between BMI increase and the risk of T2DKD, demonstrating a positive association between HBMI and an elevated risk of acquiring T2DKD in a group of 2,659 T2DM patients. This underscored the necessity of studying the burden of T2DKD in the HBMI population [10]. The global population is projected to have more than 51% classified as HBMI by 2035, which highlights a great global concern. However, there is still a gap in more comprehensive and complete research on the causal relationships and epidemiology between HBMI and T2DKD. Our study aimed to enhance our comprehension of the association between HBMI and DKD, establishing the foundation for the creation of more precise preventive treatments for patients.

With the help of Mendelian Randomization (MR), we utilized genetic variation as an instrumental variable to assess the causal relationship between exposure factors (HBMI) and disease outcomes (DKD), while being able to avoid mixed factors influencing, reduce biases [11–13]. Due to the lack of T2DKD data, we opted for the DKD dataset as an alternative. MR analysis revealed an important relationship between HBMI and DKD, furthermore, what is the current epidemiological trend of DKD attributable to HBMI? The study obtained in-depth answers from the Global Burden of Disease Study (GBD) 2021. We explored the global disease burden of T2DKD attributable to HBMI (HBMI-T2DKD) from 1990 to 2021 and made predictions on the future trend changes from 2022 to 2049. The study is expected to serve as a significant addition and expansion to prior investigations, aiding policymakers in developing more accurate preventive strategies.

## 2. Method

### 2.1. Definition

The Body Mass Index (BMI) is a quantitative measure of human body weight, determined by dividing weight in kilograms by the square of height in meters (Eq. 1). It is commonly employed as a straightforward method to diagnose obesity, owing to its simple calculation. Body weight is classified according to the BMI index: underweight (below 18.5), normal weight range (18.5–24.9), overweight (25.0–29.9), obese (greater than 30.0). The term ‘High BMI’ mentioned in this study refers to a BMI of 25 or greater [14].

(Eq. 1) $$\text{BMI}=\frac{\text{Weight }\left(\text{kg}\right)}{{\text{Height}}^{2}\;\left(m^{2}\right)}$$

### 2.2. MR analysis

#### 2.2.1. Study design

**Figure 1.**
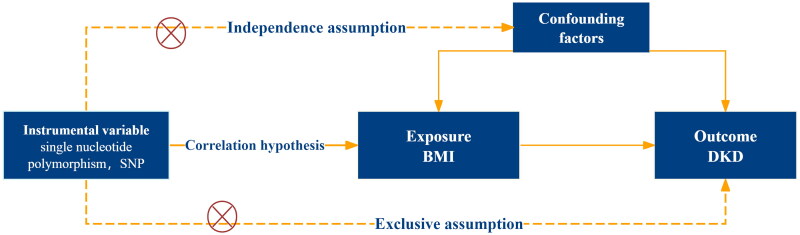
Mendelian randomization analysis thought process and assumptions. BMI, body mass index; DKD, diabetic kidney disease.

In this study, multiple single nucleotide polymorphisms (SNPs) representing genetic variation were selected as instrumental variables (IVs) and subjected to two-sample MR analysis. Summary statistics from genome-wide association studies (GWAS) were used to conduct the MR analysis to investigate the association between BMI and DKD, with BMI serving as the exposure and DKD as the outcome. All MR analyses were conducted using R version 4.2.1, with the package ‘Two-SampleMR’. The analysis was based on three key assumptions: (1) the instrumental variables were directly associated with the exposure; (2) the instrumental variables were independent of potential confounding factors; (3) the instrumental variables affected the outcome solely through the exposure (Figure 1). This study followed the STROBE-MR statement (https://www.strobe-mr.org/) (Supplementary 3).

#### 2.2.2. Data sources and SNPs selection

The datasets for BMI and DKD were obtained from the IEU database (https://gwas.mrcieu.ac.uk/), ensuring consistency in ethnicity and year [15]. The GWAS-ID for the BMI dataset is ebi-a-GCST90025994, which includes data from 457,756 European participants, collected in 2021. The GWAS-ID for the DKD dataset is ebi-a-GCST90018832, which includes data from 452,280 European participants, also from 2021. All relevant data in the IEU (OPEN GWAS) database could be open access. These samples were obtained from independent databases, ensuring minimal overlap and bias.

Appropriate SNPs were selected from these datasets to serve as instrumental variables. A P-value threshold of <5×10^-8^ was applied for screening, and an r^2^ value <0.001 with a clumping distance of 10,000 kb was used to remove linkage disequilibrium. The F-statistic was calculated to confirm a strong association between the instrumental variables and the exposure, with an F-value >10 generally indicating a robust association. Potentially confounded SNPs were excluded using the Phenoscanner database (http://www.phenoscanner.medschl.cam.ac.uk/). The minor allele frequency (MAF) for the exposure factor was set at 0.001, as the MAF affects statistical power, genetic heterogeneity, population specificity, and data quality control. The MR effect allele was set to ‘action = 3′ to define the direction of the effect of the allele on the exposure variable, thus minimizing potential issues arising from inconsistent directions and enhancing the reliability of the results.

#### 2.2.3. Statistical analysis

The inverse variance weighted (IVW) method was used as the primary analysis to estimate the causal relationship between BMI and DKD, with *p* < 0.05 indicating statistical significance. The robustness of the results was assessed by applying the weighted median method, MR-Egger regression, weighted mode method, and simple mode method. Heterogeneity was assessed using Cochran’s Q test, with *p* ≤ 0.05 indicating the presence of heterogeneity. The MR-Egger method was used to detect horizontal pleiotropy. If heterogeneity or horizontal pleiotropy existed in the results, the IVW random effects model was used; otherwise, the fixed effects model was applied. A ‘leave-one-out’ sensitivity analysis was performed to determine whether the removal of individual SNPs would significantly affect the results. Forest plots, scatter plots, and funnel plots were generated to visualize the findings.

### 2.3. Global burden analysis

#### 2.3.1. Data source

The data utilized in our analysis was obtained from the GBD 2021, which could be accessed at the following link: https://vizhub.healthdata.org/gbd-results/. The GBD 2021 was conducted by the Institute for Health Metrics and Evaluation at the University of Washington. It used a standardized and comparable method to evaluate disease burden data for 204 countries/territories, covering 371 diseases and injuries, as well as 88 risk factors worldwide [16,17]. The disease diagnoses and definitions in the GBD database primarily came from ICD-10. The Institutional Review Board at the University of Washington has granted a waiver of informed consent for the identifiable data utilized in the GBD project.

To quantify the current state of HBMI-T2DKD, we extracted data on death cases, Disability-Adjusted Life Years (DALYs), age-standardized mortality rate (ASMR), and age-standardized DALY rate (ASDR) for different sexes, age groups, GBD regions, SDI regions, and 204 countries/territories worldwide from the GBD 2021, which included a 95% uncertainty interval (UI), making the parameter estimates highly reliable. DALYs is a statistic used to measure the global disease burden of HBMI-T2DKD. It is the sum of years lived with disability (YLDs) and years of life lost (YLLs). YLD is calculated by multiplying the number of individuals with HBMI-T2DKD by the appropriate disability weights. YLL is computed by subtracting the standard life expectancy from the age at death of each individual, and aggregating these differences for all deceased individuals in the population [18]. In addition, the GBD 2021 uses the Socio-demographic Index (SDI) to measure a nation’s or region’s socioeconomic development. The GBD 2021 categorizes nations/regions worldwide into five tiers of socioeconomic development: high (>0.81), high-middle (0.70–0.81), middle (0.61–0.69), low-middle (0.46–0.60), and low (<0.46). Based on this, we could investigate the inequalities in the disease burden of HBMI-T2DKD across regions with varying levels of economic development, helping to support the establishment of more targeted prevention programs tailored to the economic conditions of each region.

#### 2.3.2. Descriptive analysis

Death cases, DALYs cases and ASMR, ASDR for HBMI-T2DKD per 100,000 population was computed to understand the global burden of HBMI-T2DKD by sexes, age groups, SDI regions, GBD regions and countries/territories. Decomposition analysis was also used to quantitatively analyze the drivers behind changes in a certain indicator. In burden of disease studies, the effect obtained from decomposition analysis refers to the impact of changes in a particular factor on the total death and DALYs when other drivers remain unchanged as the year changes [19].

#### 2.3.3. Trend analysis

The Joinpoint regression model was applied to evaluate the piecewise log-linear temporal trends of HBMI-T2DKD death and DALYs from 1990 to 2021, followed by a visual examination. The Joinpoint regression model identifies turning points in disease trends and estimates the annual percent change (APC) between these points, as well as the overall average annual percent change (AAPC). If the calculated APC/AAPC and 95% confidence interval (CI) are all greater than zero, it suggests that ASR is increasing throughout that time period. If the calculated APC/AAPC and the upper limit of its 95% CI are less than zero, it suggests that ASR is falling. If the 95% confidence interval includes zero, the ASR is considered stable [20,21].

#### 2.3.4. Predictive analysis

Based on trend changes from 1990 to 2021, we utilized the logarithmic linear age-period-cohort model to forecast the global cases and ASR of death and DALYs from 2022 to 2049. Bayesian age-period-cohort models (BAPC) are an analytical method based on Bayesian statistical methods that is commonly used to investigate the relationship between age, period, and cohort in population data. This method is mostly utilized in demography and epidemiology, and early study used it to forecast disease burden trends based on historical data [22]. BAPC employs Integrated Nested Laplace Approximations (INLA) for complete Bayesian inference.

All analyses and visualizations were conducted in R version 4.4 and Joinpoint Regression Program Version 5.0. All statistical tests were two-sided, and P-values less than 0.05 were considered statistically significant.

## 3. Results

### 3.1. MR analysis

**Figure 2.**
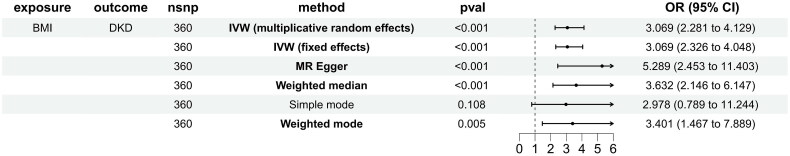
Visualization of mendelian randomization analysis results. BMI, body mass index; DKD, diabetic kidney disease; IVW, inverse variance weighted.

In this study, 360 SNPs associated with BMI were identified and utilized for subsequent MR analysis. (Supplementary 4_S1). The IVW method was employed as the primary analytical approach to assess whether HBMI was a risk factor for DKD. The Cochran’s Q heterogeneity test results from both the IVW and MR-Egger methods indicated that the selected IVs exhibited heterogeneity (Supplementary 4_S2). This heterogeneity might stem from the instrumental variables being derived from different analytical platforms, experiments, populations, and other factors. Due to the presence of heterogeneity, we chose the IVW random effects model and used MR-Egger method to process and correct for heterogeneity. Moreover, the results obtained from the IVW method, weighted median method, MR-Egger method, weighted mode method, and simple mode method were consistent, further reinforcing the robustness of our findings. (Supplementary 4_S3 and Figure 2).

Specifically, the IVW random effects model revealed a significant association between BMI and DKD [OR = 3.024, 95%CI: 2.281–4.129), *p* = 1.304 × 10^−13^ < 0.05]. OR exceeded 1 suggested that a higher BMI was associated with an increased risk of developing DKD. This association remained significant and consistent across the weighted median, MR-Egger, weighted mode, and simple mode method. These results further corroborated the hypothesis that higher BMI was associated with an increased risk of DKD. Additionally, the MR-Egger regression analysis revealed no evidence of horizontal pleiotropy (*p* = 0.0957). (Supplementary 4_S4).

The scatter plot showed that the causal direction was consistent across all methods’ results. The funnel plot demonstrated symmetry, which further supported the validity of our analysis. A leave-one-out sensitivity analysis of the IVW results, in which each SNP was removed individually, showed that no specific SNP significantly influenced the causal estimate. (Supplemental 2_Figure 1). In conclusion, the MR analysis provided strong evidence that high BMI was a risk factor for the development of DKD.

### 3.2. GBD analysis

#### 3.2.1. Overview of the global burden

**Figure 3.**
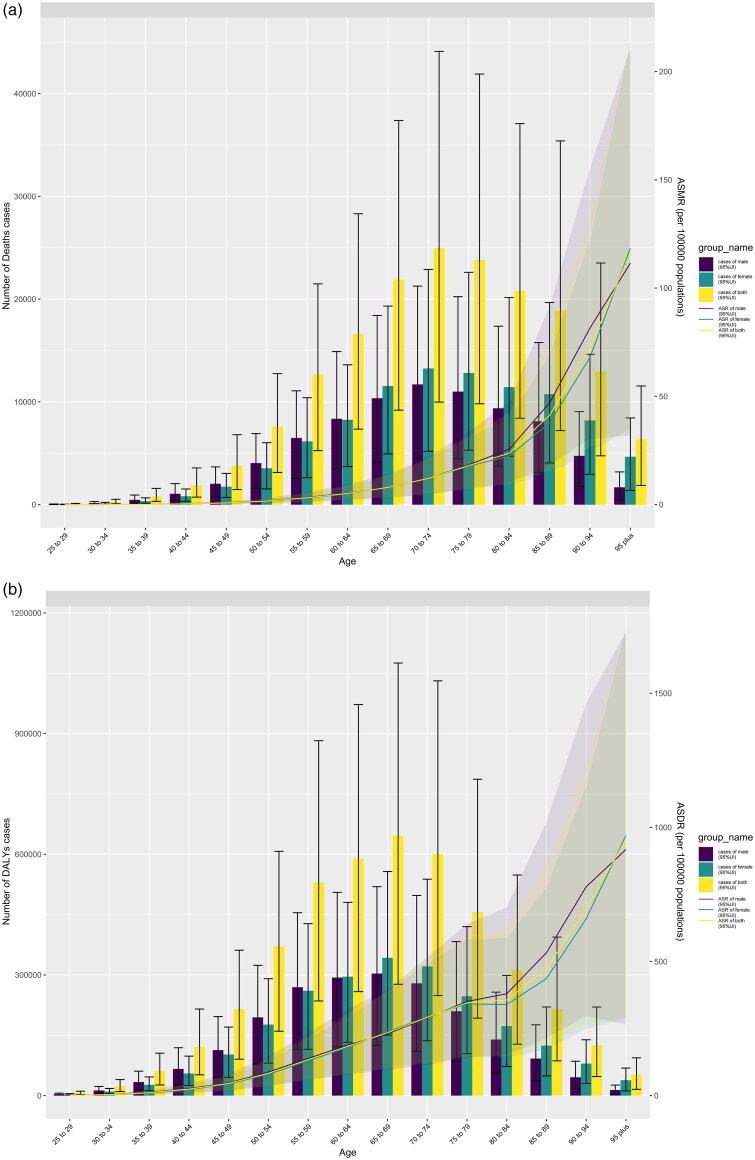
Cases and ASR of HBMI-T2DKD by different sexes and age groups in 2021. Notes: (a) Death, (b) DALYs. HBMI-T2DKD, chronic kidney disease due to diabetes mellitus type 2 attributable to high body mass index; DALYs, the Disability-Adjusted Life Years; ASMR, age-standardized mortality rate; ASDR, age-standardized DALYs rate.

Globally, the number of death cases of HBMI-T2DKD rose from 40,479 (95% UI 17,726 to 67,320) in 1990 to 173,263 (95% UI 76,311 to 288,454) in 2021, and DALYs cases rose from 1,197,972 (95% UI 539,562 to 1,925,808) in 1990 to 4,323,077 (95% UI 1,942,948 to 6,820,314) in 2021. (Figure 3, Supplemental 2_Figure 1) From 1990 to 2021, ASMR and ASDR both increased, with ASMR going from 1.16 per 100,000 population (95% UI 0.51 to 1.92) in 1990 to 2.07 per 100,000 population (95% UI 0.91 to 3.47) in 2021 and ASDR going from 30.82 per 100,000 population (95% UI 13.87 to 49.54) in 1990 to 50.14 per 100,000 population (95% UI 22.56 to 79.15) in 2021. Males outperformed females in terms of ASMR and ASDR in 2021. ASMR of males was 2.18 (95% UI 0.93 to 3.76) while ASMR of females was 2.00 (95% UI 0.90 to 3.22). ASDR of males was 51.77 (95% UI 22.66 to 85.70) while ASDR of females was 48.81 (95% UI 22.75 to 75.47) (Figure 3, Table 1, Supplemental 1_Table 1).

**Table 1.** Cases, ASR and AAPC of HBMI-T2DKD by global and different sexes, SDI region, age groups in 2021.

	Death						DALYs					
	Cases	95%UI	ASMR	95%UI	AAPC	95%CI	Cases	95%UI	ASMR	95%UI	AAPC	95%CI
**Global**	**173263**	76311 to 288454	2.07	0.91 to 3.47	**1.9**	1.78 to 2.02	4323077	1942948 to 6820314	50.14	22.56 to 79.15	1.58	1.49 to 1.68
**Sexes**												
**Males**	**79621**	33881 to 137066	2.18	0.93 to 3.76	**1.9**	1.79 to 2	2069470	905007 to 3375308	51.77	22.66 to 85.70	1.63	1.54 to 1.73
**Females**	**93642**	42092 to 150923	2	0.90 to 3.22	**1.87**	1.75 to 2	2253607	1048253 to 3491730	48.81	22.75 to 75.47	1.53	1.43 to 1.63
**SDI regions**												
**High SDI**	**50020**	22604 to 80654	2.12	0.99 to 3.37	**2.4**	2.21 to 2.58	1061064	513223 to 1612585	51.33	25.18 to 75.92	1.83	1.7 to 1.97
**High-middle SDI**	**30385**	13379 to 49247	1.56	0.69 to 2.52	**1.35**	1.23 to 1.47	758916	342841 to 1172844	38.42	17.49 to 59.21	1	0.81 to 1.2
**Low SDI**	**6576**	2434 to 12080	1.5	0.55 to 2.82	**0.99**	0.86 to 1.11	197965	76711 to 345654	38.01	14.38 to 66.68	0.89	0.79 to 0.98
**Low-middle SDI**	**25400**	10614 to 41639	1.93	0.8 to 3.23	**1.87**	1.74 to 2	722612	317973 to 1143690	49.42	21.34 to 79.15	1.87	1.71 to 2.03
**Middle SDI**	**60715**	25812 to 106474	2.46	1.05 to 4.37	**1.7**	1.47 to 1.92	1578335	687990 to 2608880	58.59	25.5 to 98.29	1.58	1.43 to 1.72
**Ages**												
**25 to 29**	**52**	14 to 118	0.01	0 to 0.02	**0.64**	0.34 to 0.94	6052	2788 to 10910	1.03	0.47 to 1.85	0.09	−0.05 to 0.23
**30 to 34**	**261**	87 to 521	0.04	0.01 to 0.09	**0.99**	0.66 to 1.32	23166	9885 to 39730	3.83	1.64 to 6.57	0.67	0.34 to 1.01
**35 to 39**	**805**	320 to 1584	0.14	0.06 to 0.28	**1.02**	0.83 to 1.21	60375	26330 to 105414	10.76	4.69 to 18.79	0.93	0.82 to 1.03
**40 to 44**	**1874**	730 to 3574	0.37	0.15 to 0.71	**1.5**	1.35 to 1.65	121167	51623 to 215176	24.22	10.32 to 43.01	1.35	1.21 to 1.5
**45 to 49**	**3786**	1473 to 6805	0.8	0.31 to 1.44	**1.62**	1.41 to 1.83	215127	90404 to 361425	45.43	19.09 to 76.33	1.45	1.24 to 1.65
**50 to 54**	**7593**	3129 to 12752	1.71	0.7 to 2.87	**1.78**	1.65 to 1.9	370833	160023 to 607210	83.35	35.97 to 136.48	1.63	1.53 to 1.73
**55 to 59**	**12638**	5254 to 21487	3.19	1.33 to 5.43	**1.87**	1.8 to 1.95	530080	235209 to 882465	133.95	59.44 to 223	1.71	1.61 to 1.82
**60 to 64**	**16617**	7348 to 28309	5.19	2.3 to 8.85	**1.93**	1.86 to 2.01	588965	258424 to 972241	184.02	80.75 to 303.78	1.7	1.61 to 1.79
**65 to 69**	**21899**	9195 to 37382	7.94	3.33 to 13.55	**1.85**	1.64 to 2.05	645907	276780 to 1075459	234.16	100.34 to 389.88	1.58	1.43 to 1.73
**70 to 74**	**24959**	9980 to 44108	12.13	4.85 to 21.43	**1.74**	1.61 to 1.87	600109	248691 to 1031280	291.54	120.82 to 501.01	1.52	1.42 to 1.62
**75 to 79**	**23817**	9816 to 41905	18.06	7.44 to 31.77	**1.86**	1.68 to 2.03	456545	192528 to 786515	346.17	145.98 to 596.37	1.54	1.41 to 1.68
**80 to 84**	**20799**	8409 to 37074	23.75	9.6 to 42.33	**1.81**	1.67 to 1.95	311847	127808 to 548413	356.06	145.93 to 626.16	1.46	1.32 to 1.59
**85 to 89**	**18862**	7203 to 35395	41.25	15.75 to 77.41	**1.99**	1.8 to 2.18	216084	86367 to 394093	472.61	188.9 to 861.94	1.57	1.41 to 1.73
**90 to 94**	**12950**	4750 to 23512	72.39	26.55 to 131.43	**2.22**	2.05 to 2.39	124803	47318 to 220169	697.64	264.5 to 1230.72	1.88	1.69 to 2.07
**95 plus**	**6350**	1873 to 11550	116.51	34.37 to 211.92	**2.58**	2.39 to 2.78	52017	15499 to 93814	954.39	284.36 to 1721.26	2.49	2.3 to 2.67

(HBMI-T2DKD, chronic kidney disease due to diabetes mellitus type 2 attributable to high body mass index; DALYs, the Disability-Adjusted life years; ASMR, age-standardized mortality rate; ASDR, age-standardized DALYs rate; AAPC, average annual percent change; SDI, socio-demographic index).

From 1990 to 2021, The population with heavy disease burden was the older adults. In 2021, The age group with the highest ASMR and ASDR was 95 and above (ASMR: 116.51, 95% UI 34.37 to 211.92; ASDR: 954.39, 95% UI 284.36 to 1721.26) (Figure 3).

**Figure 4.**
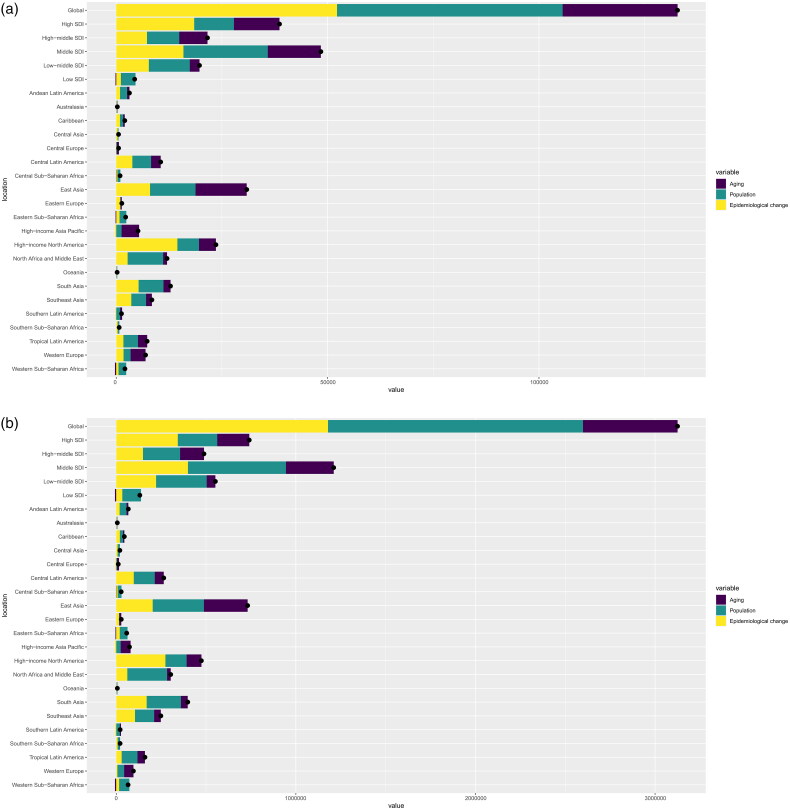
Changes in death (a) and DALYs (b) according to population-level determinants of population growth, aging, and epidemiological change from 1990 to 2021 at the global level by different SDI regions. HBMI-T2DKD, chronic kidney disease due to diabetes mellitus type 2 attributable to high body mass index; DALYs, the Disability-Adjusted Life Years; SDI, Socio-demographic Index.

In terms of GBD regions, East Asia had the highest number of death and DALYs cases. Population growth and aging contributed a lot to the increase in HBMI-T2DKD burden. (Figure 4) In 2021, the region with the highest ASMR was Andean Latin America (6.9, 95% UI 2.93 to 11.19), while the region with the highest ASDR was Central Latin America (125.48, 95% UI 62.43 to 190.67) (Table 1, Supplemental 1_Tables 2 and 3).

Middle SDI region had the highest disease burden in both 1990 and 2021. In 2021, the ASMR of middle SDI region was 2.46 (95% UI 1.05 to 4.37) and the ASDR was 58.59 (95% UI 25.5 to 98.29). In middle SDI region, population growth and epidemiological change were the key driver of the increase in HBMI-T2DKD death and DALYs. However, the low SDI region had the lowest disease burden in 2021, with ASMR of 1.5 (95% UI 0.55 to 2.82) and an ASDR of 38.01 (95% UI 14.38 to 66.68) (Supplemental 1_Figure 2, Supplemental 1_Figure 3).

In terms of national data, the top five countries/territories with the highest ASMR in 2021 were American Samoa, the Northern Mariana Islands, Nauru, Niue, and Fiji. The top five nations with the highest ASDR were American Samoa, Nauru, the Northern Mariana Islands, Niue, and the Federated States of Micronesia (Figure 5).

Figure 5.Global burden and trends of HBMI-T2DKD by different countries/territories in 2021.Notes: (a) ASMR, (b) ASDR, (c) AAPC of death, (d) AAPC of DALYs. HBMI-T2DKD, chronic kidney disease due to diabetes mellitus type 2 attributable to high body mass index; DALYs, the Disability-Adjusted Life Years; ASMR, age-standardized mortality rate; ASDR, age-standardized DALYs rate; AAPC, average annual percent change.

**Figure:**
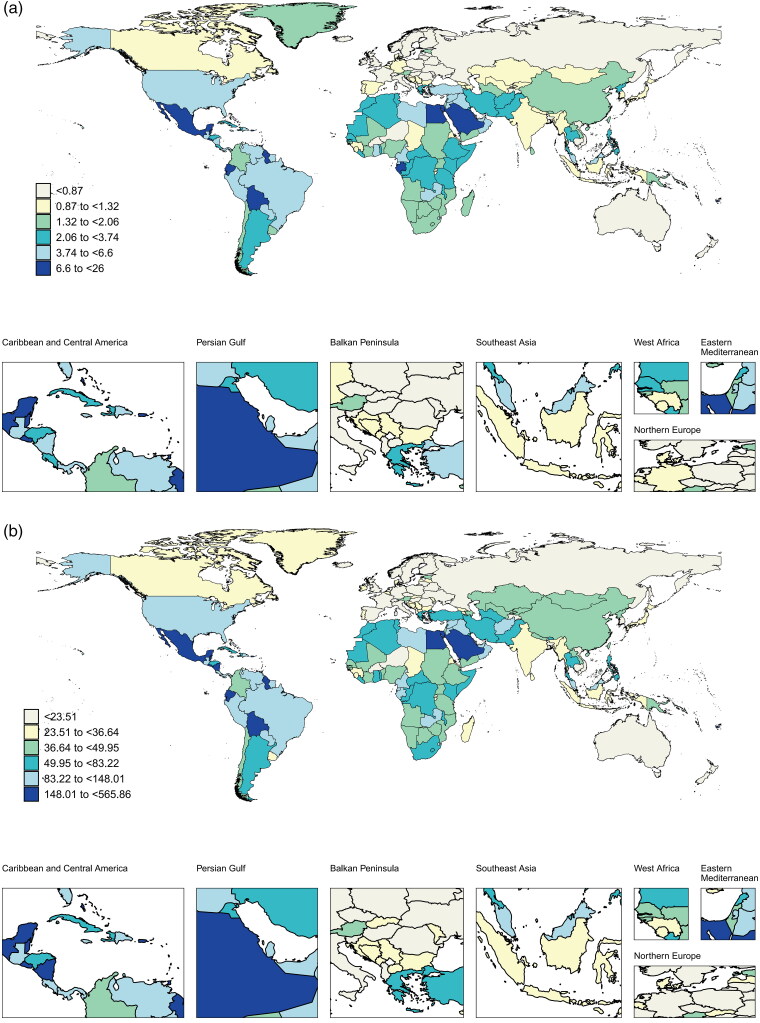

**Figure:**
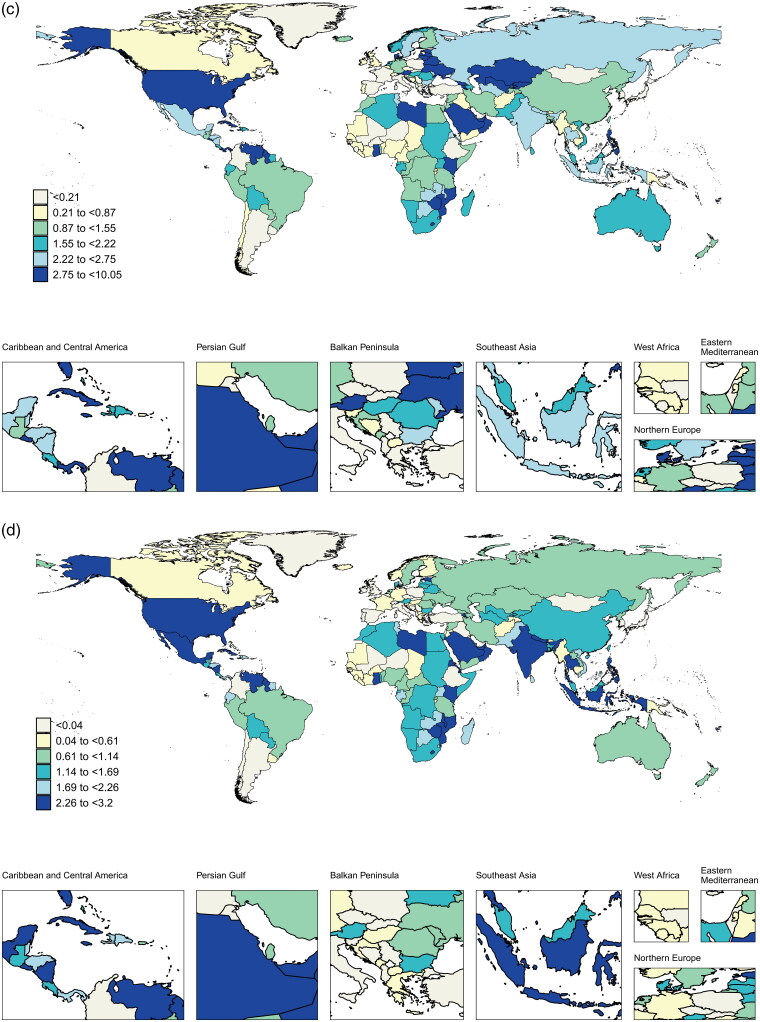

#### 3.2.2. Trend analysis

Globally, from 1990 to 2021, the AAPC of death and DALYs was 1.9 (95%CI 1.78 to 2.02) and 1.58 (95%CI 1.49 to 1.68), respectively, indicating an increasing trend. The growth rate of ASDR was discovered to vary between time periods. In terms of the global APC of DALYs, the ASDR rose rather quickly between 1996–2003 (APC = 2.64), 2007–2016 (APC = 1.66), and 2019–2021 (APC = 1.65), whereas growth was slower between 2016–2019 (APC = 0.40). The greatest rise in APC of death occurred between 1997–2000 (APC = 3.79) and 2000–2003 (APC = 2.84). However, the APC of death has slowed in recent years, dropping from 2.13 in 2007–2015 to 1.11 in 2015–2021 (Figure 6).

Figure 6.Trends of HBMI-T2DKD by different sexes (a. death, b. DALYs), SDI regions (c. death, d. DALYs) from 1990 to 2021, calculated by joinpoint regression.HBMI-T2DKD, chronic kidney disease due to diabetes mellitus type 2 attributable to high body mass index; DALYs, the Disability-Adjusted Life Years; AAPC, average annual percent change; APC, average annual percent change.

**Figure:**
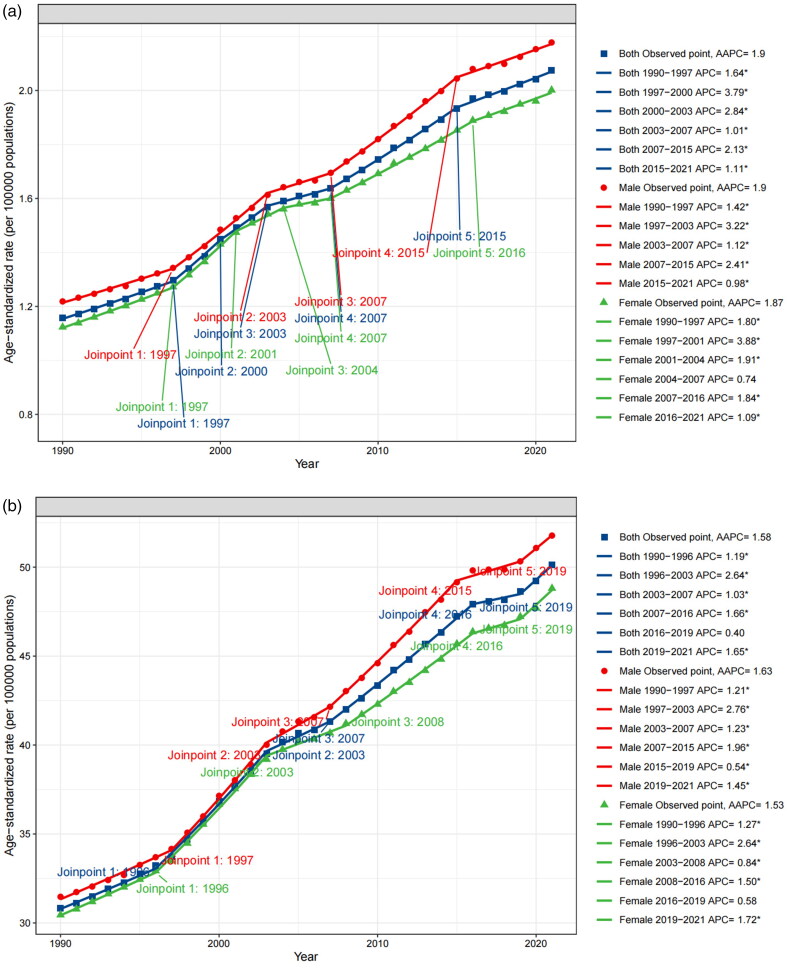

**Figure:**
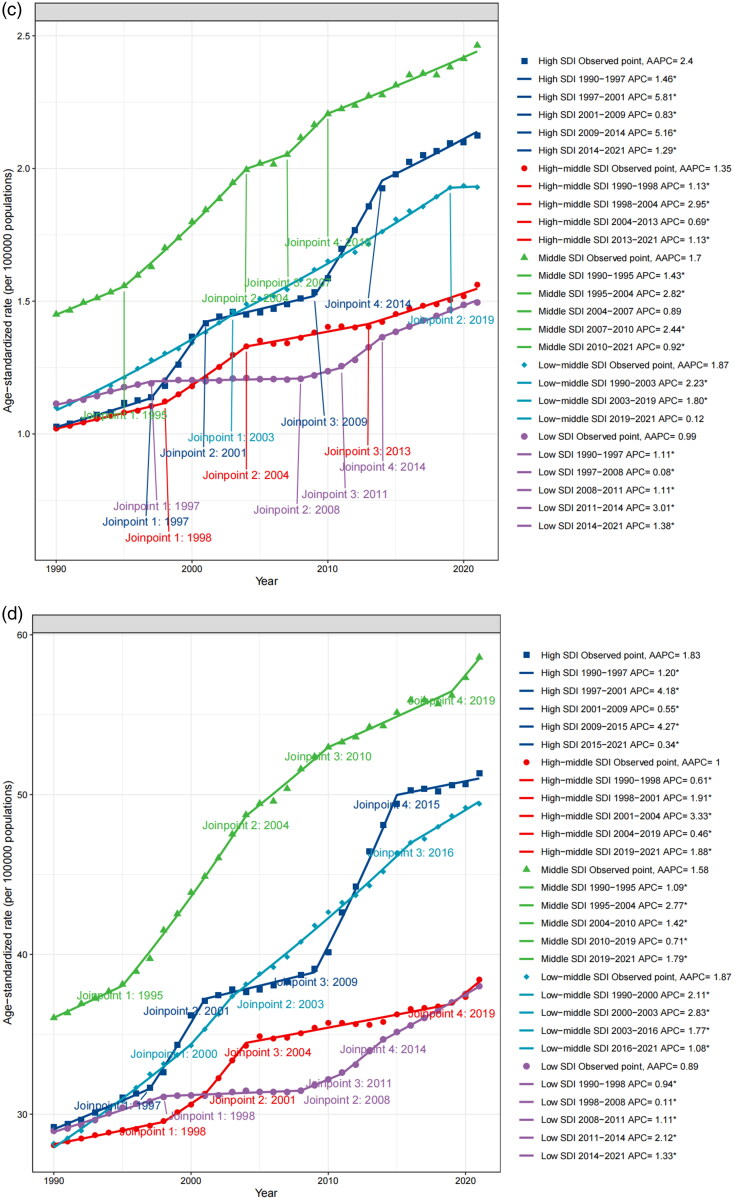

In terms of sexes, males experienced faster growth in disease burden (AAPC of ASMR = 1.90, 95% CI 1.79 to 2.00; AAPC of ASDR = 1.63, 95% CI 1.54 to 1.73) than females (AAPC of ASMR = 1.87, 95% CI 1.75 to 2.00; AAPC of ASDR = 1.53, 95% CI 1.43 to 1.63). The trend growth changed in both males and females at various yearly intervals were broadly aligned with the global patterns reported for both sexes (Figure 6).

The older adults continued to have the fastest growth in disease burden. The three age groups with the fastest-growing ASDR were 95 and above (AAPC = 2.49, 95% CI 2.3 to 2.67), 90 to 94 (AAPC = 1.88, 95% CI 1.69 to 2.07), and 55 to 59 (AAPC = 1.71, 95% CI 1.61 to 1.82). The three age groups with the fastest-growing ASMR were 95 and above (AAPC = 2.58, 95% CI 2.39 to 2.78), 90 to 94 (AAPC = 2.22, 95% CI 2.05 to 2.39), and 85 to 89 (AAPC = 1.99, 95% CI 1.8 to 2.18), followed closely by 55 to 59 and 60 to 64. The disease burden had also increased significantly among those aged 55 to 64. Meanwhile, between 1990 and 2021, the ASMR and ASDR for those under 40 remained relatively stable, while those over 90 had a steady increase (Supplemental 1_Figure 5).

ASDR and ASMR growth rates varied by SDI regions. In terms of death, the region with the highest AAPC was high SDI region (AAPC = 2.4, 95% CI 2.21 to 2.58), while low SDI region had the lowest (AAPC = 0.99, 95% CI 0.86 to 1.11). In terms of DALYs, the highest AAPC was low-middle SDI region (AAPC = 1.87, 95% CI 1.71 to 2.03), while the lowest was low SDI region (AAPC = 0.89, 95% CI 0.79 to 0.98) (Figure 6).

Differences in the AAPC of death and DALYs among nations were also interesting. Twenty-five countries/territories had an AAPC of death smaller than 0, with 95% CI containing zero. Poland had the lowest AAPC of deaths (–2.15, 95% CI −2.83 to −1.46), whereas the three highest were Ukraine, Armenia, and Estonia. Furthermore, thirty-three countries/territories had an AAPC of DALYs smaller than 0, indicating that their ASDR had remained relatively steady for the past 22 years. Poland had the lowest AAPC of DALYs (–1.71, 95% CI −2.22 to −1.19). The top three countries/territories with the highest AAPC of DALYs were American Samoa, El Salvador, and Kenya (Supplemental 1_Table 4).

#### 3.2.3. Predictive analysis

Between 2022 and 2049, it was expected that worldwide death cases, DALYs cases, ASMR, ASDR would all rise significantly. By 2049, the cases of death and DALYs caused by HBMI-T2DKD was anticipated to be 597,495 (95% CI 0 to 1,311,513) and 12,925,045 (95% CI 0 to 26,876,904). Concurrently, global ASMR and ASDR for HBMI-T2DKD were expected to climb to 3.17 (95% CI 0 to 6.96) and 77.32 (95% CI 0 to 160.85). By 2049, ASMR and ASDR were expected to be greater in males (ASMR: 2.83, 95% CI −0.04 to 5.70; ASDR: 69.63, 95% CI 3.75 to 135.50) than in females (ASMR: 2.66, 95% CI −0.20 to 5.52; ASDR: 67.40, 95% CI 1.94 to 132.86), in line with rates in 2021. The anticipated disease burden across different age groups by 2049 had received special attention. The older population continued to bear the largest disease burden, warranting major attention. By 2049, the lowest ASMR and ASDR were expected in the 25 to 29 age group (ASMR = 0.01, 95% CI 0 to 0.03, ASDR = 1.07, 95% CI 0 to 2.49), while the highest were in the 95 and above group (ASMR = 200.00, 95% CI 0 to 440.56, ASDR = 1756.03, 95% CI 0 to 3654.49) (Figure 7, Supplemental 1_Table 5).

Figure 7.Projection of HBMI-T2DKD by different sexes and ages from 2022 to 2049. (a) Death by sex, (b) Death by age, (c) DALYs by sex, (d) DALYs by age. HBMI-T2DKD, chronic kidney disease due to diabetes mellitus type 2 attributable to high body mass index; DALYs, the Disability-Adjusted life years.

**Figure:**
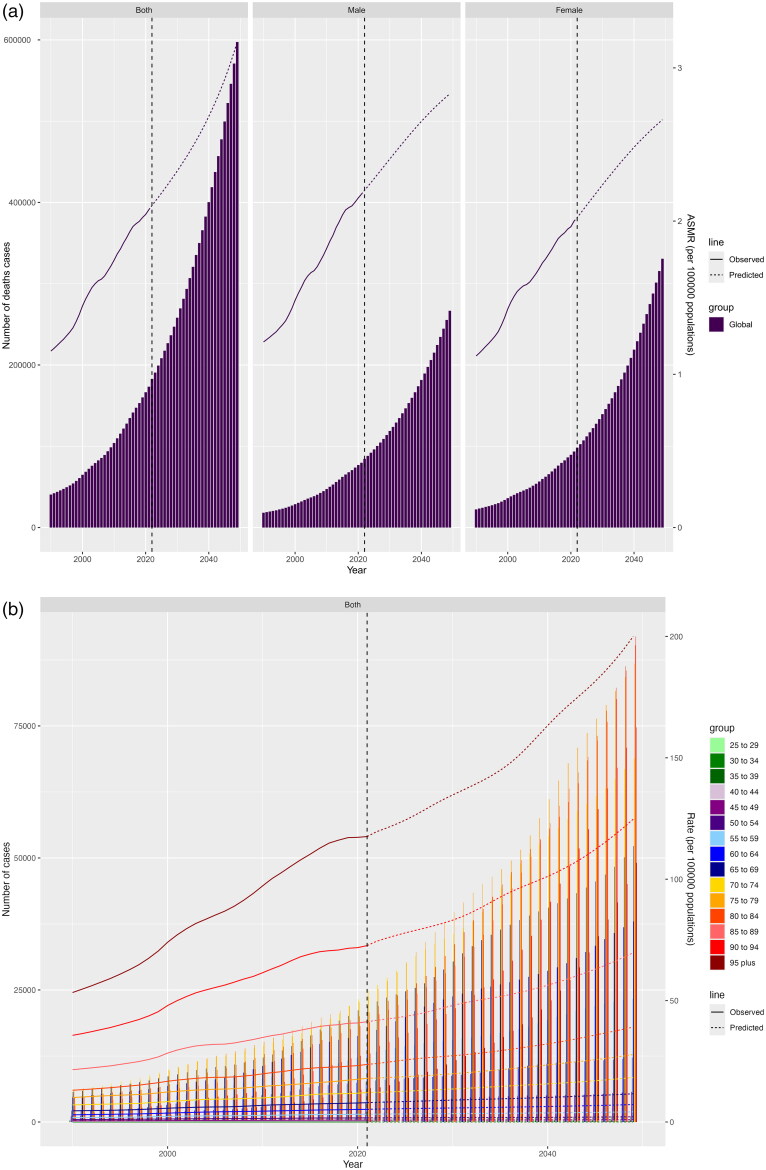

**Figure:**
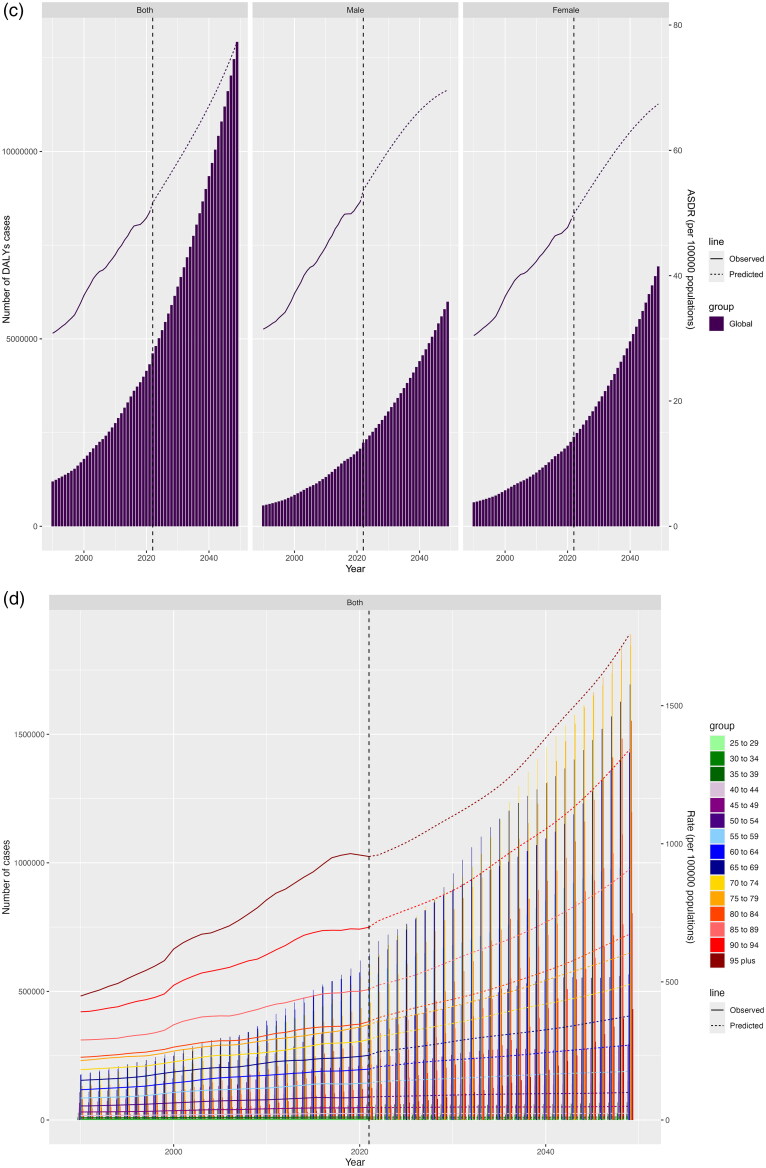

## 4. Discussion

The study first employed the MR analysis to identify a significant causal relationship between BMI and DKD, which aligned with the conclusions of existing observational and experimental studies. Specifically, the study indicated that HBMI significantly increased the risk of DKD, while effectively controlling for confounding factors. These results provided a foundation for subsequent secondary observational studies based on the GBD 2021 database. It was undeniable that the epidemiological study based on the GBD database was unable to fully control for potential confounding factors, nor did it establish a definitive causal relationship between risk factors and diseases. To address these limitations, this study employed a two-sample MR analysis to confirm the positive correlation between BMI and DKD, making it more comprehensive and robust compared to previous GBD-related studies. On the other hand, the MR analysis method was theoretically an analytical method with causal relationships and required further validation through subsequent studies. Moreover, the database used in MR analysis only included European individuals, which raised questions about its applicability to other populations.

Although the population representativeness of MR analysis is limited, the association between HBMI and DKD has been further validated in previous studies of populations from other regions. A multi-ethnic Asian study incorporating meta-analysis revealed that overweight and obese individuals exhibited significantly higher susceptibility to DKD compared to underweight or normal-weight counterparts [23]. Furthermore, clinical research conducted in the Americas demonstrated that short-term intensive weight loss interventions substantially improved renal function in DKD patients [24]. Moreover, an Australian population-based investigation identified obesity as an independent risk factor for DKD pathogenesis [25]. These collective findings underscored the universal relevance of our discoveries across diverse global populations. To further elucidate these associations, we conducted a comprehensive analysis utilizing the GBD database to investigate disparities in disease burden related to HBMI-T2DKD across different demographic groups worldwide, thereby advancing our understanding of this critical public health issue.

The study analyzed the global disease burden of HBMI-T2DKD from 1990 to 2021. In 2021, the death cases, DALYs cases, ASMR, and ASDR linked to HBMI-T2DKD continued to rise. Over the 32 year period, the AAPC of both death and DALYs was more than zero. While the increase in cases could be attributed to worldwide population expansion, increases in ASR and AAPC showed that the global disease burden of HBMI-T2DKD was steadily rising and demanded immediate attention. At the same time, through careful analysis, we found some points that need attention and exploration.

The global disease burden on the older adults is always heavy and requires our full attention, and the burden on the middle-aged also cannot be neglected. Individuals aged 85 years and above have the highest number of death cases, DALYs cases, ASMR and ASDR. Older adults with HBMI-T2DKD have a higher likelihood of having other illnesses such as hypertension, hyperlipidemia, and heart disease [26]. These circumstances exacerbate the progression of T2DKD [27]. Sarcopenia, frailty, malnutrition, and cognitive dysfunction contribute to a harmful cycle with older adults’ diabetes and its complications [28,29]. This not only makes it more challenging to manage exercise and monitor blood sugar in older diabetic patients, but also leads to a continuous increase in BMI due to extended bed rest and lack of physical activity [30]. Consequently, this worsens the progression of T2DKD. Hence, it is advisable to regularly monitor the risk factors of older patients and study weight reduction approaches and BMI control indicators specifically tailored for the older adults. This will help reduce kidney and cardiovascular endpoint events, improve the quality of life for older adults, and simultaneously lower the overall economic burden on society to some extent. Over the past few years, the prevalence of overweight and obesity among middle-aged persons has risen due to smoking, alcohol consumption, and unhealthy lifestyle choices [31]. During middle age, there is a significant decline in health and body function [32], which is influenced by factors such as increased BMI, osteoarthritis, cardiovascular illnesses, and depressive symptoms [33], all of which pose higher risks. Furthermore, the absence of proper lifestyle management increases the vulnerability of middle-aged individuals with HBMI to diseases such as T2DM, cardiovascular disease, and some types of cancer [34]. To effectively address the needs of individuals with HBMI-T2DKD in this age group, it is crucial to emphasize lifestyle management and actively encourage weight loss. Additionally, it is important to closely monitor blood sugar and kidney function in individuals with HBMI, detect and intervene in T2DKD as early as possible, in order to prevent the development of end-stage kidney disease (ESKD).

The disease burden differs between SDI regions, GBD regions and countries/territories, thus we should concentrate on disease-burdened areas and the underlying reasons. According to GBD regions and countries/territories, the top five countries/territories with the highest ASMR and ASDR between 1990 and 2021 were Pacific Island nations with small geographical areas, poor economic growth, and weak healthcare systems. There are insufficient resources to maintain stable glycemic control, as well as poor monitoring for risk factors, which contributes to the early onset of T2DM complications such as DKD. Meanwhile, the low-middle SDI region have the highest AAPC of DALYs. A study have pointed out that T2DM has a particularly strong and direct economic impact on the livelihoods of people in low-income environments [35]. These regions lack sufficient economic investment in diabetes patients, and the relatively insufficient healthcare security and social investment make high-risk patients more likely to develop complications. Therefore, we emphasize the importance of establishing global health partnerships to provide financial and technical assistance to economically underdeveloped regions, which will benefit to reduce health inequities and improve the management of HBMI-T2DKD. Additionally, from the perspective of SDI regions, in terms of death and DALYs, middle SDI region had the highest disease burden in 2021, which should not be overlooked. Rapid industrialization has resulted in a significant quantity of low-cost ultra-processed foods [36], exposing people, particularly children, to environments rich in calories, fat, sugar, and salt on a daily basis. As a result, there has been a clear shift toward younger persons with high BMI, as well as a steady rise in the incidence of T2DM, increasing the burden of T2DKD. According to a poll [37], middle SDI region have the highest proportion of HBMI adolescents in the globe. The dual burden of malnutrition (DBM), which comprises both undernutrition and overnutrition, along with unequal economic growth, places a considerable strain on healthcare resources, both financial and human [38]. Because of this, early-stage T2DM patients with HBMI receive less attention, causing HBMI-T2DKD to emerge earlier. As a result, limiting the amount of processed food that enters the market while focusing on early childhood nutrition program restrictions is critical.

According to our forecasts, disease burdens will continue to rise across the globe by 2049, affecting all age groups. This not only severely affects health, but also imposes a heavy economic burden globally. One study showed that the total cost per year for each T2DKD patient in the United States was approximately $25,000 [39]. Another study revealed that in Turkey, the top three diseases with the highest direct healthcare costs related to obesity-associated comorbidities were chronic kidney disease, heart failure, and type 2 diabetes [40]. Stronger public health interventions are required to reduce the considerable economic and medical burdens created by HBMI-T2DKD worldwide. One of the most important things that can do to stop T2DKD from getting worse and to slow down DKD’s progression is to control the BMI [41]. Dietary and exercise modifications are still critical lifestyle adjustments [42], particularly for developing nations and regions. Actively monitoring risk factors is another effective way to lower kidney end-point occurrences in older HBMI-T2DKD patients. Education and public awareness, early screening and diagnosis, standardized diabetes treatment, targeted lifestyle interventions, healthcare infrastructure development, policy support, and community participation are all critical components of public health strategies. Poland recommends that overweight or obese people should be tested for T2DM once a year [43], with an emphasis on early detection. Whole grains are the main staple of the Icelandic diet [44]. Studies have shown that whole grains can effectively lower metabolic waste products such as phosphorus, urea, and creatinine while also slowing glucose absorption, and the dietary fiber they contain may lower the glycemic index [45]. A generally healthy diet pattern reduces the prevalence of HBMI-T2DKD diseases. Since 1922, the Norwegian government has charged a sugar tax, and a 2019 study found that the country’s per capita sugar consumption has decreased to its lowest level in 44 years [46]. A diet rich in deep-sea fish [47], the country’s comprehensive healthcare system and a love of skiing are all factors that reduce the disease burden of HBMI-T2DKD. These can provide us with reference. Additionally, recent study indicated that GLP-1 receptor agonists, SGLT-2 inhibitor, and weight loss surgery can all benefit HBMI-T2DKD while protecting kidney function [42,48–51]. It is vital to investigate anti-obesity medications in CKD in order to make more discoveries and find solutions [52].

However, several limitations should be acknowledged. First, the quality and scope of the data within the GBD 2021 may affect the accuracy of our findings, potentially leading to underreporting and misdiagnosis of HBMI-T2DKD, particularly in low-income regions. We hope that in the future, closer international cooperation can strengthen the monitoring of major chronic diseases across regions and implement standardized diagnoses to improve data quality. Second, due to limitations in the GBD 2021, detailed information, such as the incidence and prevalence of HBMI-T2DKD, is not available, which introduces a degree of bias into our analysis. We look forward to more in-depth research in the future that integrates multiple epidemiological indicators to comprehensively show the disease burden of HBMI-T2DKD. Third, since the data in the GBD database is aggregated, it lacks more individual information about the surveyed subjects. Therefore, we are unable to conduct a comprehensive analysis of other risk factors and covariates, such as blood pressure, blood lipids, lifestyle, economic status, and healthcare coverage, that may affect the disease burden of HBMI-T2DKD, which would allow for a better consideration of the potential confounding variables’ impact on the results. In the future, we will conduct research by integrating multiple databases and real-world cohorts to analyze the impact of other covariates on disease burden and prediction models. Fourth, the prediction results regarding future changes in disease burden lack further exploration of the effects of interventions, policy changes, and other factors. Using independent datasets for external validation or comparing with the results of multiple predictive models are feasible measures to improve the accuracy of predictions in the future. In addition, in the MR analysis, the lack of T2DKD-specific datasets necessitates reliance on a broader DKD dataset, which may limit the precision of conclusions and bring potential uncertainty to causal inference. Moreover, the MR analysis is conducted solely based on European populations. In summary, we hope to address these shortcomings as much as possible through the combined analysis of GBD and MR, and to clarify the relationship between HBMI and DKD from different perspectives.

## 5. Conclusion

This study explored the association between HBMI and DKD based on the GBD 2021 and MR analysis. It highlights that HBMI remains a significant risk factor for the development of DKD, and the disease burden of HBMI-T2DKD has been steadily increasing from 1990 to 2021, with projections indicating a continued rise through 2049, particularly among older adults and in previously identified regions. Therefore, more stringent and targeted weight loss programs, along with more proactive monitoring and management of DKD, are essential for alleviating the worldwide disease burden and advancing public health.

## Supplementary Material

Supplemental Material

Online Supplementary Material 4.xlsx

Online Supplementary Material 2.docx

Online Supplementary Material 3.docx

## Data Availability

The original contributions presented in the study are included in the article/Supplementary Material. Further inquiries can be directed to the corresponding authors.

## References

[1] de Boer IH. Kidney disease and related findings in the diabetes control and complications trial/epidemiology of diabetes interventions and complications study. Diabetes Care. 2014;37(1):24–30. doi: 10.2337/dc13-2113. 24356594 PMC3867994

[2] Afkarian M, Zelnick LR, Hall YN, et al. Clinical manifestations of kidney disease among US adults with diabetes, 1988-2014. JAMA. 2016;316(6):602–610. doi: 10.1001/jama.2016.10924. 27532915 PMC5444809

[3] International Diabetes Federation. Diabetes and kidney disease. IDF diabetes atlas. Brussels, Belgium: International Diabetes Federation; 2021.

[4] Kim MK, Kim DM. Current status of diabetic kidney disease and latest trends in management. J Diabetes Investig. 2022;13(12):1961–1962. doi: 10.1111/jdi.13895. PMC9720214 36001045

[5] American Diabetes Association Professional Practice Committee. 1. Improving care and promoting health in populations: standards of Care in Diabetes-2024. Diabetes Care. 2024;47(Suppl 1):11–19. doi: 10.2337/dc24-S001. PMC10725798 38078573

[6] Tuttle KR, Bakris GL, Bilous RW, et al. Diabetic kidney disease: a report from an ADA Consensus Conference. Diabetes Care. 2014;37(10):2864–2883. doi: 10.2337/dc14-1296. 25249672 PMC4170131

[7] Xie Y, Bowe B, Mokdad AH, et al. Analysis of the Global Burden of Disease study highlights the global, regional, and national trends of chronic kidney disease epidemiology from 1990 to 2016. Kidney Int. 2018;94(3):567–581. doi: 10.1016/j.kint.2018.04.011. 30078514

[8] Jiang W, Wang J, Shen X, et al. Establishment and validation of a risk prediction model for early diabetic kidney disease based on a Systematic Review and Meta-Analysis of 20 Cohorts. Diabetes Care. 2020;43(4):925–933. doi: 10.2337/dc19-1897. 32198286

[9] Liu C, Zhang J, Wei X, et al. Effects of sleep duration and changes in body mass index on diabetic kidney disease: a prospective cohort study. Front Endocrinol (Lausanne). 2023;14:1278665. doi: 10.3389/fendo.2023.1278665. 37964958 PMC10641014

[10] Sun Z, Wang K, Yun C, et al. Correlation between the variability of different obesity indices and diabetic kidney disease: a retrospective cohort study based on populations in Taiwan. Diabetes Metab Syndr Obes. 2023;16:2791–2802. doi: 10.2147/DMSO.S425198. 37720422 PMC10504903

[11] Suzuki S, Goto A, Nakatochi M, et al. Body mass index and colorectal cancer risk: a Mendelian randomization study. Cancer Sci. 2021;112(4):1579–1588. doi: 10.1111/cas.14824. 33506574 PMC8019210

[12] Wang L, Cao W, Xi MH, et al. Appendectomy and the risk of neurodegenerative diseases: a two-samPle Mendelian randomization study. Asian J Surg. 2024;47(1):673–674. doi: 10.1016/j.asjsur.2023.09.170. 37806880

[13] Qing J, Li Y, Soliman KM, et al. A practical guide for nephrologist peer reviewers: understanding and appraising Mendelian randomization studies. Ren Fail. 2025;47(1):2445763. doi: 10.1080/0886022X.2024.2445763. 39806780 PMC11734392

[14] WHO Consultation on Obesity (‎1999: Geneva, Switzerland)‎ & World Health Organization. Obesity: preventing and managing the global epidemic. Report of a WHO consultation. World Health Organ Tech Rep Ser. 2000;894:i–253. 11234459

[15] Lyon MS, Andrews SJ, Elsworth B, et al. The variant call format provides efficient and robust storage of GWAS summary statistics. Genome Biol. 2021;22(1):32. doi: 10.1186/s13059-020-02248-0. 33441155 PMC7805039

[16] GBD 2021 Diseases and Injuries Collaborators. Global incidence, prevalence, years lived with disability (YLDs), disability-adjusted life-years (DALYs), and healthy life expectancy (HALE) for 371 diseases and injuries in 204 countries and territories and 811 subnational locations, 1990-2021: a systematic analysis for the Global Burden of Disease Study 2021. Lancet. 2024;403(10440):2133–2161. doi: 10.1016/S0140-6736(24)00757-8. 38642570 PMC11122111

[17] GBD 2021 Risk Factors Collaborators. Global burden and strength of evidence for 88 risk factors in 204 countries and 811 subnational locations, 1990-2021: a systematic analysis for the Global Burden of Disease Study 2021. Lancet. 2024;403(10440):2162–2203. doi: 10.1016/S0140-6736(24)00933-4. Correction appears in: lancet. 2024;404(10449):244. doi: 10.1016/S0140-6736(24)01458-2. 38762324 PMC11120204

[18] Murray CJ, Vos T, Lozano R, et al. Disability-adjusted life years (DALYs) for 291 diseases and injuries in 21 regions, 1990-2010: a systematic analysis for the Global Burden of Disease Study 2010. Lancet. 2012;380(9859):2197–2223. doi: 10.1016/S0140-6736(12)61689-4. Correction appears in Lancet. 2013;381(9867):628. 23245608

[19] Xie D, Ma T, Cui H, et al. Global burden and influencing factors of chronic kidney disease due to type 2 diabetes in adults aged 20-59 years, 1990-2019. Sci Rep. 2023;13(1):20234. doi: 10.1038/s41598-023-47091-y. 37981642 PMC10658077

[20] Kim HJ, Fay MP, Feuer EJ, et al. Permutation tests for joinpoint regression with applications to cancer rates. Statist Med. 2000;19(3):335–351. Erratum in: stat Med. 2001;20(4):655. doi: 10.1002/(sici)1097-0258(20000215)19:3<335::aid-sim336>3.0.co;2-z. 10649300

[21] Li HZ, Du LB. Application of Joinpoint regression model in cancer epidemiological time trend analysis. Zhonghua Yu Fang Yi Xue Za Zhi. 2020;54(8):908–912. doi: 10.3760/cma.j.cn112150-20200616-00889. 32842323

[22] Liu J, Liu M, Chai Z, et al. Projected rapid growth in diabetes disease burden and economic burden in China: a spatio-temporal study from 2020 to 2030. Lancet Reg Health West Pac. 2023;33:100700. doi: 10.1016/j.lanwpc.2023.100700. 36817869 PMC9932123

[23] Man REK, Gan ATL, Fenwick EK, et al. The relationship between generalized and abdominal obesity with diabetic kidney disease in type 2 diabetes: a multiethnic Asian study and meta-analysis. Nutrients. 2018;10(11):1685. doi: 10.3390/nu10111685. 30400648 PMC6266073

[24] Friedman AN, Chambers M, Kamendulis LM, et al. Short-term changes after a weight reduction intervention in advanced diabetic nephropathy. Clin J Am Soc Nephrol. 2013;8(11):1892–1898. doi: 10.2215/CJN.04010413. 23929927 PMC3817909

[25] Sukkar L, Kang A, Hockham C, et al. EXTEND45 study steering committee, incidence and associations of chronic kidney disease in community participants with diabetes: a 5-year prospective analysis of the EXTEND45 study. Diabetes Care. 2020;43(5):982–990. doi: 10.2337/dc19-1803. 32161053 PMC7809711

[26] American Diabetes Association Professional Practice Committee. 10. Cardiovascular disease and risk management: standards of Care in Diabetes-2024. Diabetes Care. 2024;47(Suppl 1):179–218. doi: 10.2337/dc24-S010. PMC10725811 38078592

[27] Leehey DJ, Zhang JH, Emanuele NV, et al. BP and renal outcomes in diabetic kidney disease: the Veterans Affairs Nephropathy in Diabetes Trial. Clin J Am Soc Nephrol. 2015;10(12):2159–2169. doi: 10.2215/CJN.02850315. 26482258 PMC4670761

[28] Remelli F, Maietti E, Abete P, et al. Prevalence of obesity and diabetes in older people with sarcopenia defined according to EWGSOP2 and FNHI criteria. Aging Clin Exp Res. 2022;34(1):113–120. doi: 10.1007/s40520-021-01949-1. 34398439 PMC8795057

[29] Kirkman MS, Briscoe VJ, Clark N, et al. Diabetes in older adults: a consensus report. J Am Geriatr Soc. 2012;60(12):2342–2356. doi: 10.1111/jgs.12035. 23106132 PMC4525769

[30] Trim WV, Walhin JP, Koumanov F, et al. The impact of physical inactivity on glucose homeostasis when diet is adjusted to maintain energy balance in healthy, young males. Clin Nutr. 2023;42(4):532–540. doi: 10.1016/j.clnu.2023.02.006. 36857962

[31] NCD Risk Factor Collaboration (NCD-RisC). Worldwide trends in underweight and obesity from 1990 to 2022: a pooled analysis of 3663 population-representative studies with 222 million children, adolescents, and adults. Lancet. 2024;403(10431):1027–1050. doi: 10.1016/S0140-6736(23)02750-2. 38432237 PMC7615769

[32] Lachman ME, Teshale S, Agrigoroaei S. Midlife as a pivotal period in the life course: balancing growth and decline at the crossroads of youth and old age. Int J Behav Dev. 2015;39(1):20–31. doi: 10.1177/0165025414533223. 25580043 PMC4286887

[33] Solomon DH, Colvin A, Lange-Maia BS, et al. Factors associated with 10-year declines in physical health and function among women during midlife. JAMA Netw Open. 2022;5(1):e2142773. doi: 10.1001/jamanetworkopen.2021.42773. 35006247 PMC8749479

[34] Jia G, Shu XO, Liu Y, et al. Association of adult weight gain with major health outcomes among middle-aged Chinese persons with low body weight in early adulthood. JAMA Netw Open. 2019;2(12):e1917371. doi: 10.1001/jamanetworkopen.2019.17371. 31834393 PMC6991199

[35] Seuring T, Archangelidi O, Suhrcke M. The economic costs of type 2 diabetes: a global systematic review. Pharmacoeconomics. 2015;33(8):811–831. doi: 10.1007/s40273-015-0268-9. 25787932 PMC4519633

[36] Baker P, Machado P, Santos T, et al. Ultra-processed foods and the nutrition transition: global, regional and national trends, food systems transformations and political economy drivers. Obes Rev. 2020;21(12):e13126. doi: 10.1111/obr.13126. 32761763

[37] World Obesity Federation. World Obesity Atlas 2024. London: World Obesity Federation; 2024.

[38] Gogas Yavuz D, Akhtar O, Low K, et al. The economic impact of obesity in Turkey: a micro-costing analysis. Clinicoecon Outcomes Res. 2024;16:123–132. doi: 10.2147/CEOR.S446560. 38476579 PMC10929251

[39] Folkerts K, Petruski-Ivleva N, Kelly A, et al. Annual health care resource utilization and cost among type 2 diabetes patients with newly recognized chronic kidney disease within a large U.S. administrative claims database. J Manag Care Spec Pharm. 2020;26(12):1506–1516. doi: 10.18553/jmcp.2020.26.12.1506. 33251992 PMC10391265

[40] Kiosia A, Dagbasi A, Berkley JA, et al. The double burden of malnutrition in individuals: identifying key challenges and re-thinking research focus. Nutr Bull. 2024;49(2):132–145. doi: 10.1111/nbu.12670. 38576109

[41] Jacob P, McCafferty K. Assessment and management of chronic kidney disease in people living with obesity. Clin Med (Lond). 2023;23(4):353–356. doi: 10.7861/clinmed.2023-0195. 37524431 PMC10541045

[42] ElSayed NA, Aleppo G, Bannuru RR, American Diabetes Association Professional Practice Committee., et al. Summary of Revisions: standards of Care in Diabetes-2024. Diabetes Care. 2024;47(Supplement_1):S5–S10. doi: 10.2337/dc24-SREV. 38078579 PMC10725800

[43] Araszkiewicz A, Bandurska-Stankiewicz E, Budzyński A, et al. 2019 Guidelines on the management of diabetic patients. A position of Diabetes Poland. Clin Diabetol. 2019;8(1):1–95. doi: 10.5603/DK.2019.0001.

[44] Steingrímsdóttir L, Thorkelsson G, Eythórsdóttir E. Chapter 6 - Food, nutrition, and health in Iceland. In: Andersen V, Bar E, Wirtanen G, editors. In: Elsevier Traditional and Ethnic Food Series, Nutritional and health aspects of food in nordic countries. London (UK): Academic Press; 2018. p. 145–177. doi: 10.1016/B978-0-12-809416-7.00006-8.

[45] Develaraja S, Reddy A, Yadav M, et al. Whole grains in amelioration of metabolic derangements. J Nutrit Health Food Sci. 2016;4(4):1–11. doi: 10.15226/jnhfs.2016.00173. PMC5609487 28944285

[46] Henley J. Sweet spot: norwegians cut sugar intake to lowest level in 44 years. The Guardian; 2019. Nov 20. [accessed 2025 Mar 3]. https://www.theguardian.com/world/2019/nov/20/norwegians-cut-sugar-intake-to-lowest-level-in-44-years

[47] Zhang TT, Xu J, Wang YM, et al. Health benefits of dietary marine DHA/EPA-enriched glycerophospholipids. Prog Lipid Res. 2019;75:100997. doi: 10.1016/j.plipres.2019.100997. 31442526

[48] Colhoun HM, Lingvay I, Brown PM, et al. Long-term kidney outcomes of semaglutide in obesity and cardiovascular disease in the SELECT trial. Nat Med. 2024;30(7):2058–2066. doi: 10.1038/s41591-024-03015-5. 38796653 PMC11271413

[49] Toyama T, Neuen BL, Jun M, et al. Effect of SGLT2 inhibitors on cardiovascular, renal and safety outcomes in patients with type 2 diabetes mellitus and chronic kidney disease: a systematic review and meta-analysis. Diabetes Obes Metab. 2019;21(5):1237–1250. doi: 10.1111/dom.13648. 30697905

[50] Martin-Taboada M, Vila-Bedmar R, Medina-Gómez G. From obesity to chronic kidney disease: how can adipose tissue affect renal function? Nephron. 2021;145(6):609–613. doi: 10.1159/000515418. 33882488

[51] Serra A, Granada ML, Romero R, et al. The effect of bariatric surgery on adipocytokines, renal parameters and other cardiovascular risk factors in severe and very severe obesity: 1-year follow-up. Clin Nutr. 2006;25(3):400–408. doi: 10.1016/j.clnu.2005.11.014. 16709438

[52] Taber-Hight E, Gilmore A, Friedman AN. Anti-obesity pharmacotherapy in adults with chronic kidney disease. Kidney Int. 2024;105(2):269–280. doi: 10.1016/j.kint.2023.10.014. 37926421

