# Supplementary material for: Causal impact of elevated body mass index on diabetic kidney disease: an integrated Mendelian randomization and Global Burden of Disease Study 2021 analysis
Source: Ren Fail. 2025 Mar 17;47(1):2472981. doi: 10.1080/0886022X.2025.2472981 (PMC11984565; doi:10.1080/0886022X.2025.2472981)
Supplement: Supplemental Material [file IRNF_A_2472981_SM9356.pdf]

Online Supplementary Material 1

Title: Causal Impact of Elevated Body Mass Index on Diabetic Kidney Disease: An Integrated Mendelian Randomization and Global Burden of Disease 2021 Analysis

Authors: Ye-xin Chen †, Dong-sen Hu †, Mao-xuan Lin, Zi-heng Gao, Han-zhang Hong, Yu-xin Hu, Ling-zi Yao, Gai-wen Cui, Lin Wang*

Fig. S1 Cases and ASR of HBMI-T2DKD by different sexes and ages in 1990.
Notes: (A) Death, (B) DALYs.
ASR, age-standardized rate; HBMI-T2DKD, chronic kidney disease due to diabetes mellitus type 2 attributable to high body mass index; DALYs, the Disability-Adjusted Life Years; ASMR, age-standardized mortality rate; ASDR, age-standardized DALYs rate.

Fig. S2 The global ASMR(A) and ASDR(B) of HBMI-T2DKD across GBD regions by SDI.
Notes: (A) Death, (B) DALYs
HBMI-T2DKD, chronic kidney disease due to diabetes mellitus type 2 attributable to high body mass index; DALYs, the Disability-Adjusted Life Years; ASMR, age-standardized mortality rate; ASDR, age-standardized DALYs rate; SDI, Socio-demographic Index.

Fig. S3 The global ASMR(A) and ASDR(B) of HBMI-T2DKD across countries/territories by SDI.
Notes: (A) Death, (B) DALYs
HBMI-T2DKD, chronic kidney disease due to diabetes mellitus type 2 attributable to high body mass index; DALYs, the Disability-Adjusted Life Years; ASMR, age-standardized mortality rate; ASDR, age-standardized DALYs rate; SDI, Socio-demographic Index.

Fig. S4 Global burden of HBMI-T2DKD by different countries/territories in 1990.
Notes: (A) ASMR, (B) ASDR
HBMI-T2DKD, chronic kidney disease due to diabetes mellitus type 2 attributable to high body mass index; DALYs, the Disability-Adjusted Life Years; ASMR, age-standardized mortality rate; ASDR, age-standardized DALYs rate.

Fig. S5 Trends of HBMI-T2DKD by ages from 1990 to 2021, calculated by Joinpoint regression.
Notes: (A) Death, (B) DALYs
HBMI-T2DKD, chronic kidney disease due to diabetes mellitus type 2 attributable to high body mass index; DALYs, the Disability-Adjusted Life Years; AAPC, average annual percent change; APC, annual percent change.

Table S1 Cases and ASR of HBMI-T2DKD by global and different sexes, SDI regions, age groups in 1990.
ASR, age-standardized rate; HBMI-T2DKD, chronic kidney disease due to diabetes mellitus type 2 attributable to high body mass index; SDI, Socio-demographic Index; DALYs, the Disability-Adjusted Life Years; ASMR, age-standardized mortality rate; ASDR, age-standardized DALYs rate; AAPC, average annual percent change.

Table S2 Cases, ASR and AAPC of death of HBMI-T2DKD by different GBD regions from 1990 to 2021.

ASR, age-standardized rate; AAPC, average annual percent change; DALYs, the Disability-Adjusted Life Years; HBMI-T2DKD, chronic kidney disease due to diabetes mellitus type 2 attributable to high body mass index; ASMR, age-standardized mortality rate.

Table S3 Cases, ASR and AAPC of DALYs of HBMI-T2DKD by different GBD regions in 1990-2021.

ASR, age-standardized rate; AAPC, average annual percent change; DALYs, the Disability-Adjusted Life Years; HBMI-T2DKD, chronic kidney disease due to diabetes mellitus type 2 attributable to high body mass index; ASDR, age-standardized DALYs rate.

Table S4 Cases, ASR and AAPC of death and DALYs of HBMI-T2DKD by different countries/territories in 1990-2021.

ASR, age-standardized rate; AAPC, average annual percent change; DALYs, the Disability-Adjusted Life Years; HBMI-T2DKD, chronic kidney disease due to diabetes mellitus type 2 attributable to high body mass index; ASMR, age-standardized mortality rate; ASDR, age-standardized DALYs rate.

Table S5 Projection of HBMI-T2DKD from 2022 to 2049.

HBMI-T2DKD, chronic kidney disease due to diabetes mellitus type 2 attributable to high body mass index; DALYs, the Disability-Adjusted Life Years; ASMR, age-standardized mortality rate; ASDR, age-standardized DALYs rate.

A

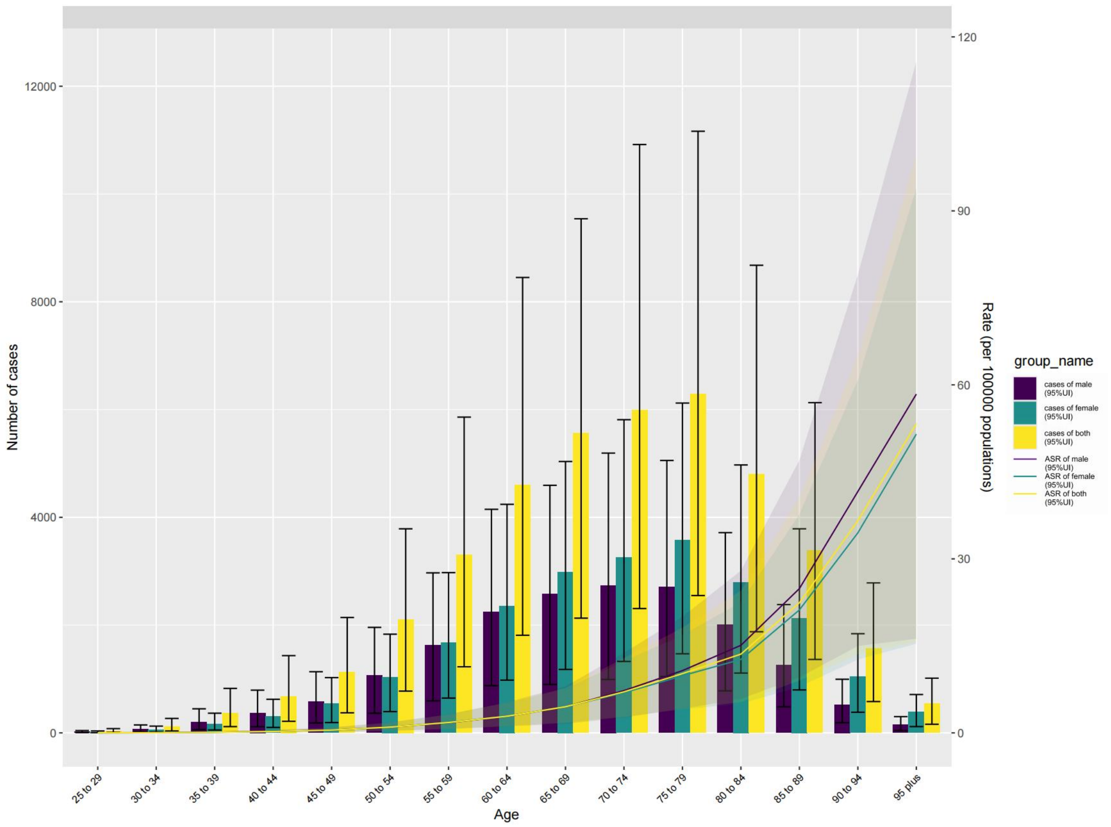

B

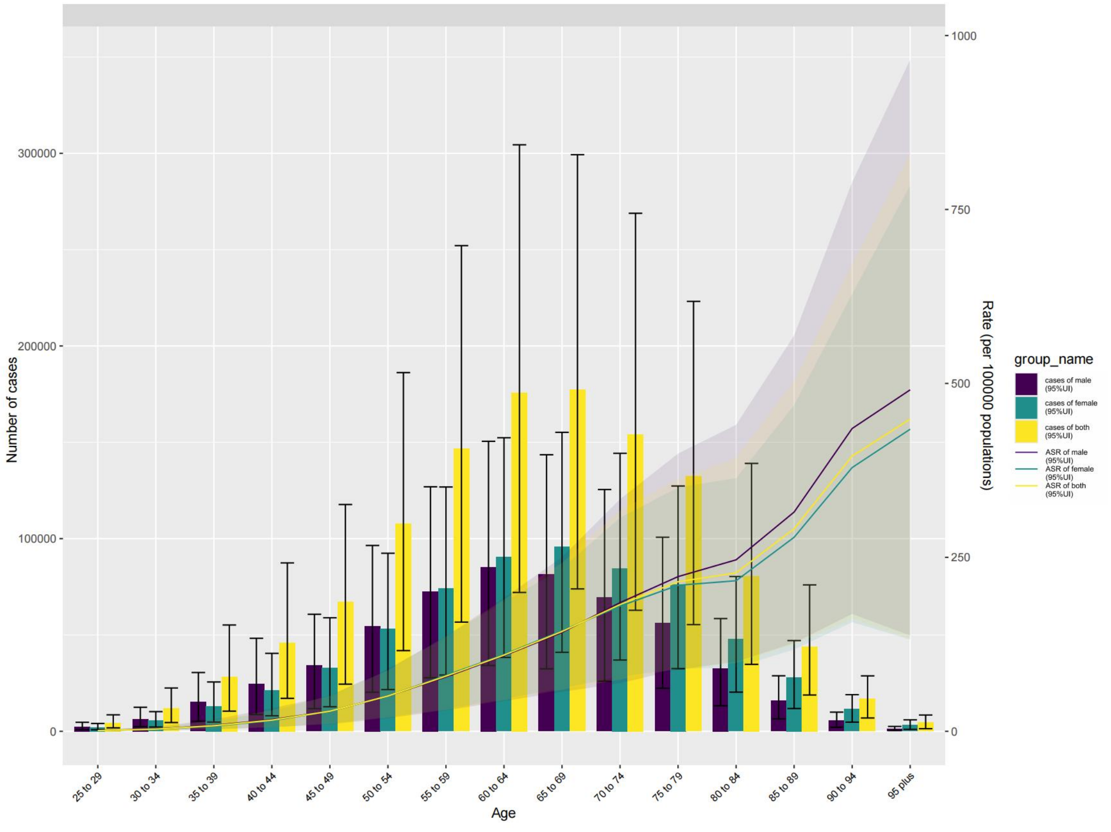

Fig. S1 Cases and ASR of HBMI-T2DKD by different sexes and ages in 1990.
Notes: (A) Death, (B) DALYs.

ASR, age-standardized rate; HBMI-T2DKD, chronic kidney disease due to diabetes mellitus type 2 attributable to high body mass index; DALYs, the Disability-Adjusted Life Years; ASMR, age-standardized mortality rate; ASDR, age-standardized DALYs rate.

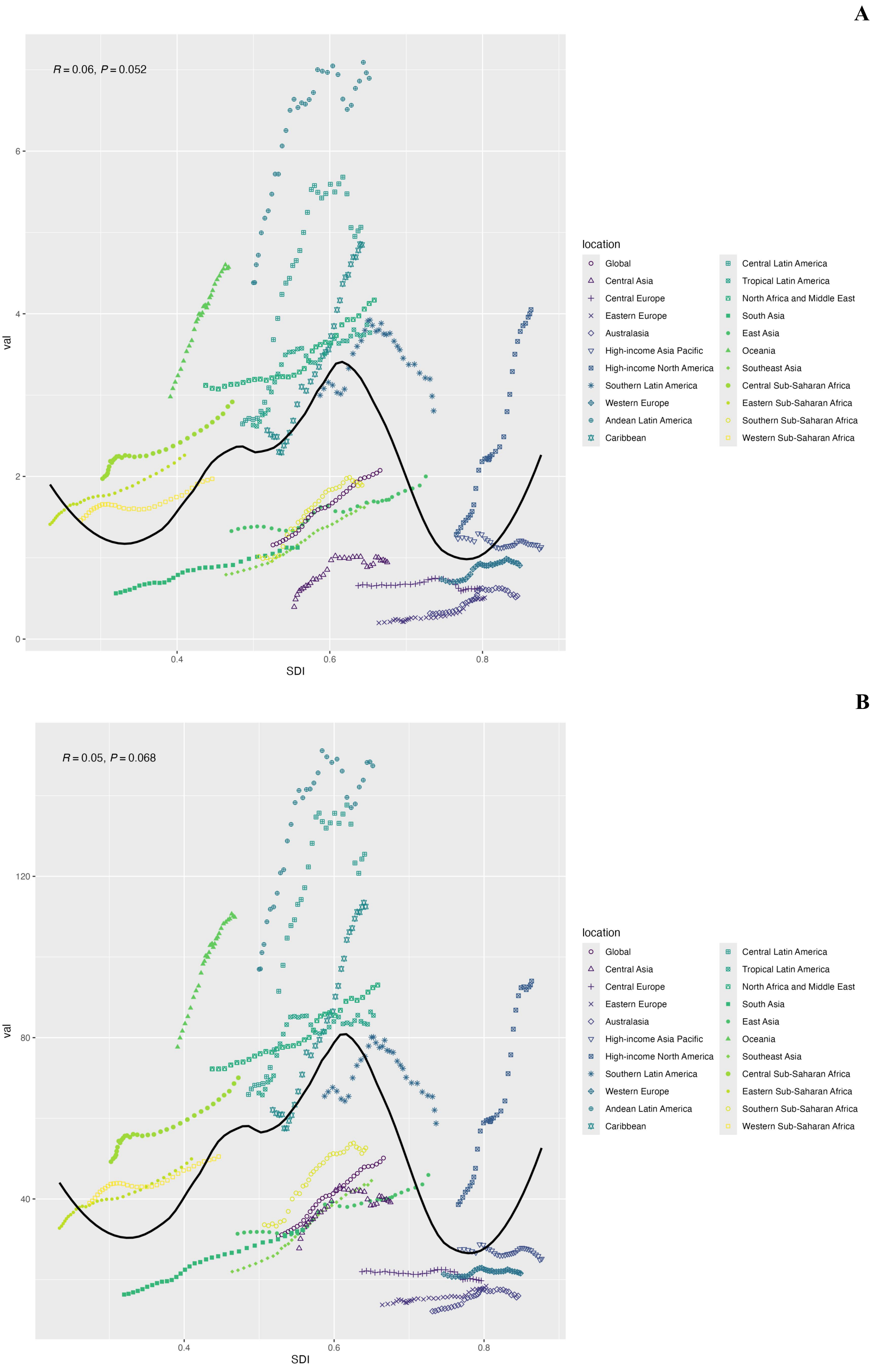

Fig. S2 The global ASMR(A) and ASDR(B) of HBMI-T2DKD across GBD regions by SDI.

Notes: (A) Death, (B) DALYs

HBMI-T2DKD, chronic kidney disease due to diabetes mellitus type 2 attributable to high body mass index; DALYs, the Disability-Adjusted Life Years; ASMR, age-standardized mortality rate; ASDR, age-standardized DALYs rate; SDI, Socio-demographic Index.

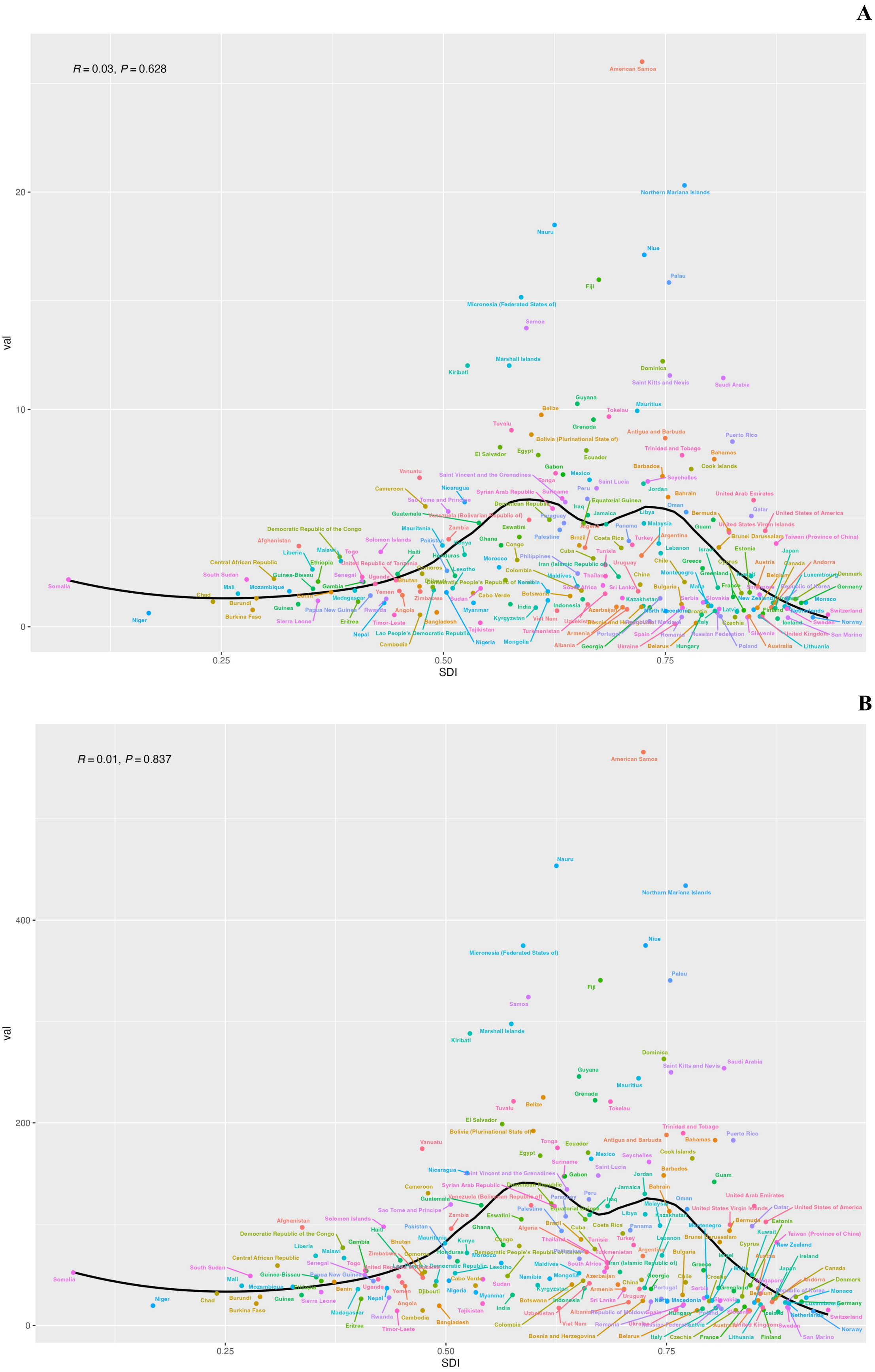

Fig. S3 The global ASMR(A) and ASDR(B) of HBMI-T2DKD across countries/territories by SDI.

Notes: (A) Death, (B) DALYs

HBMI-T2DKD, chronic kidney disease due to diabetes mellitus type 2 attributable to high body mass index; DALYs, the Disability-Adjusted Life Years; ASMR, age-standardized mortality rate; ASDR, age-standardized DALYs rate; SDI, Socio-demographic Index.

A

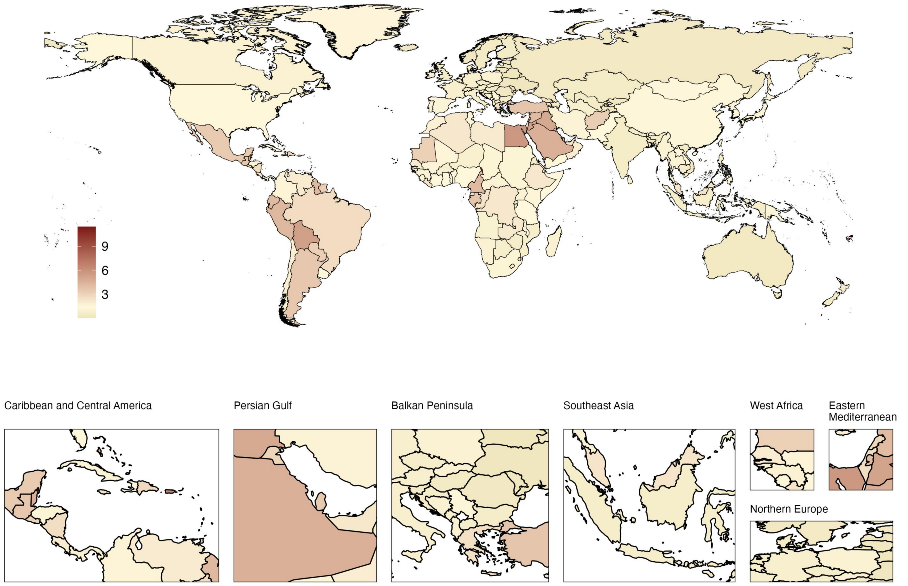

B

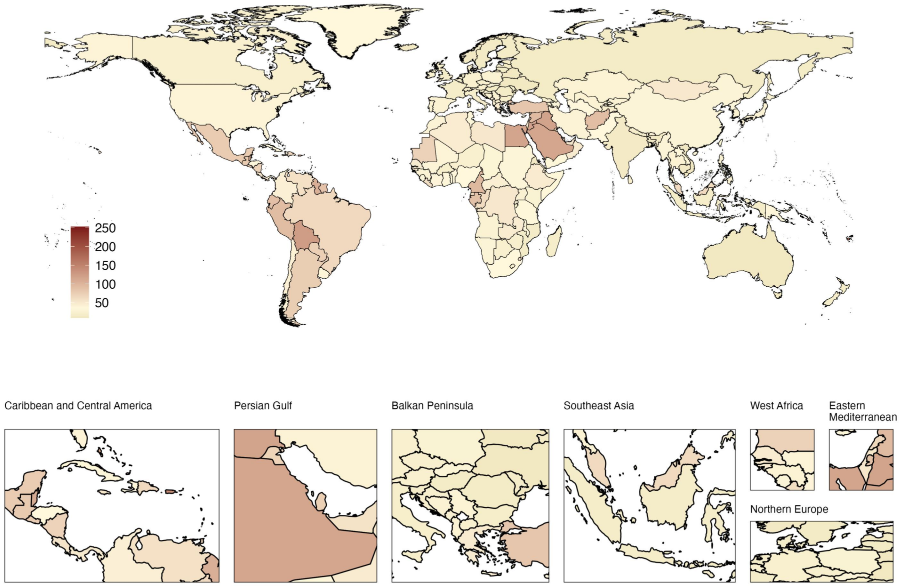

Fig. S4 Global burden of HBMI-T2DKD by different countries/territories in 1990.

Notes: (A) ASMR, (B) ASDR

HBMI-T2DKD, chronic kidney disease due to diabetes mellitus type 2 attributable to high body mass index; DALYs, the Disability-Adjusted Life Years; ASMR, age-standardized mortality rate; ASDR, age-standardized DALYs rate.

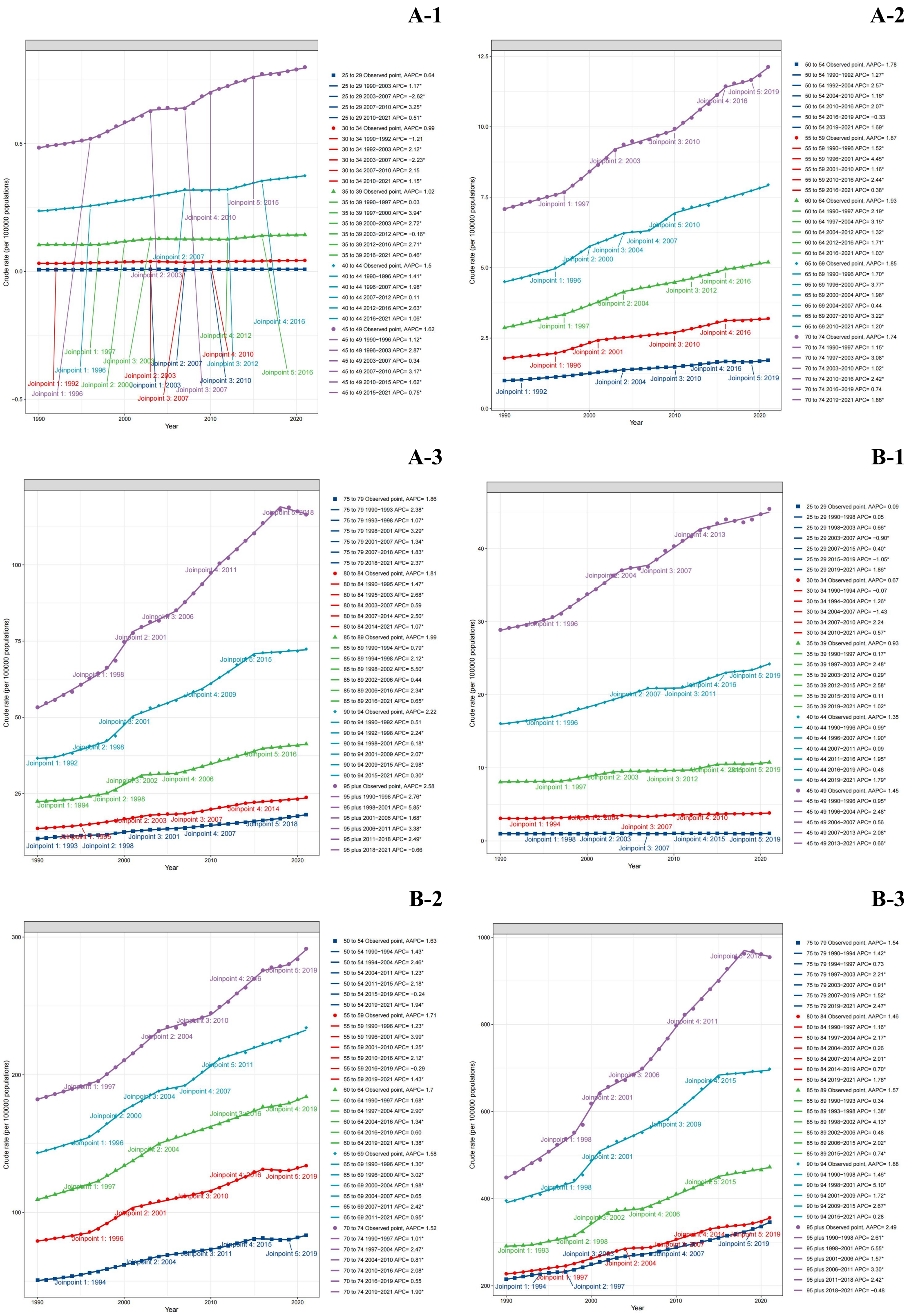

Fig. S5 Trends of HBMI-T2DKD by ages from 1990 to 2021, calculated by Joinpoint regression.

Notes: (A) Death, (B) DALYs

HBMI-T2DKD, chronic kidney disease due to diabetes mellitus type 2 attributable to high body mass index; DALYs, the Disability-Adjusted Life Years; AAPC, average annual percent change; APC, annual percent change.

	Death		DALYs	
	Cases (95% UI)	ASMR (95% UI)	Cases (95% UI)	ASDR (95% UI)
**Global**	40479 (17726to67320)	1.16 (0.51to1.92)	1197972 (539562to1925808)	30.82 (13.87to49.54)
**Sexes**				
male	18141 (7570to31336)	1.22 (0.52to2.09)	557967 (242064to925038)	31.47 (13.64to52.44)
female	22337 (9884to35884)	1.12 (1.80to0.50)	640005 (291543to1002393)	30.45 (13.87to47.53)
**SDI regions**				
High SDI	11393 (5328to17103)	1.03 (0.49to1.54)	320657 (153049to467969)	29.19 (14.14to42.27)
High-middle SDI	8800 (3877to13955)	1.02 (0.45to1.61)	270596 (123746to414614)	28.06 (12.88to42.6)
Low SDI	2218 (833to4443)	1.11 (0.42to2.24)	66781 (26221to127319)	28.96 (11.2to56.52)
Low-middle SDI	5708 (2294to9639)	1.1 (0.45to1.88)	170703 (71977to284145)	28.16 (11.72to46.86)
Middle SDI	12309 (5059to22998)	1.45 (0.6to2.75)	367740 (155641to649560)	36.03 (15.18to65.02)
**Ages**				
25 to 29	32 (8to79)	0.01 (0to0.02)	4401 (1786 to 8542)	0.99 (0.4 to 1.93)
30 to 34	123 (35to268)	0.03 (0.01to0.07)	11955 (4543 to 22469)	3.1 (1.18 to 5.83)
35 to 39	366 (116to825)	0.1 (0.03to0.23)	28382 (10458 to 55181)	8.06 (2.97 to 15.67)
40 to 44	680 (215to1433)	0.24 (0.07to0.5)	46046 (17097 to 87411)	16.07 (5.97 to 30.51)
45 to 49	1125 (372to2141)	0.48 (0.16to0.92)	67053 (24473 to 117709)	28.88 (10.54 to 50.69)
50 to 54	2106 (775to3787)	0.99 (0.36to1.78)	107795 (41871 to 186183)	50.71 (19.7 to 87.59)
55 to 59	3301 (1226to5860)	1.78 (0.66to3.16)	146772 (56669 to 252079)	79.25 (30.6 to 136.11)
60 to 64	4602 (1811to8451)	2.87 (1.13to5.26)	175767 (72056 to 304428)	109.44 (44.86 to 189.55)
65 to 69	5564 (2129to9541)	4.5 (1.72to7.72)	177240 (73910 to 299293)	143.39 (59.79 to 242.13)
70 to 74	5991 (2307to10918)	7.08 (2.73to12.9)	154203 (62810 to 268879)	182.14 (74.19 to 317.59)

75 to 79	6287 (2548to11165)	10.21 (4.14to18.14)	132322 (55373 to 223151)	214.96 (89.96 to 362.52)
80 to 84	4802 (1876to8678)	13.57 (5.3to24.53)	80546 (34743 to 139046)	227.69 (98.21 to 393.05)
85 to 89	3389 (1362to6128)	22.43 (9.02to40.55)	43973 (18839 to 75986)	291 (124.67 to 502.85)
90 to 94	1567 (581to2783)	36.58 (13.56to64.94)	16950 (6910 to 28763)	395.54 (161.25 to 671.23)
95 plus	542 (160to1015)	53.28 (15.68to99.68)	4568 (1358 to 8449)	448.67 (133.34 to 829.89)

Table S1 Cases and ASR of HBMI-T2DKD by global and different sexes, SDI regions, age groups in 1990.

ASR, age-standardized rate; HBMI-T2DKD, chronic kidney disease due to diabetes mellitus type 2 attributable to high body mass index; SDI, Socio-demographic Index; DALYs, the Disability-Adjusted Life Years; ASMR, age-standardized mortality rate; ASDR, age-standardized DALYs rate; AAPC, average annual percent change.

GBD regions	1990		2021		AAPC
	Cases (95% UI)	ASMR (95% UI)	Cases (95% UI)	ASMR (95% UI)	(95% CI)
Andean Latin America	812 (306to1336)	4.38 (1.64to7.27)	3938 (1682to6305)	6.9 (2.93to11.19)	1.52 (1.02 to 2.02)
Australasia	69 (36to99)	0.32 (0.16to0.45)	325 (161to477)	0.53 (0.27to0.78)	1.64 (1.03 to 2.26)
Caribbean	619 (257to1060)	2.57 (1.07to4.42)	2643 (1100to4369)	4.85 (2.02to7.98)	2.09 (1.54 to 2.64)
Central Asia	179 (86to261)	0.39 (0.19to0.59)	720 (374to1085)	0.94 (0.48to1.45)	2.8 (1.59 to 4.02)
Central Europe	931 (469to1336)	0.66 (0.33to0.94)	1480 (732to2376)	0.62 (0.31to0.99)	-0.15 (-0.48 to 0.18)
Central Latin America	1911 (843to3178)	2.65 (1.14to4.57)	12418 (5868to19475)	5.06 (2.39to8)	2.13 (1.58 to 2.69)
Central Sub-Saharan Africa	366 (137to710)	1.97 (0.72to3.72)	1270 (467to2385)	2.92 (1.04to5.64)	1.25 (1.09 to 1.41)
East Asia	8853 (3190to18825)	1.33 (0.49to2.77)	39725 (16343to73073)	2 (0.81to3.67)	1.3 (0.88 to 1.72)
Eastern Europe	542 (256to797)	0.2 (0.09to0.29)	1859 (948to2603)	0.51 (0.26to0.71)	3.13 (2.07 to 4.21)
Eastern Sub-Saharan Africa	906 (314to1963)	1.41 (0.5to3.1)	3135 (1153to5897)	2.26 (0.83to4.37)	1.53 (1.42 to 1.63)
High-income Asia Pacific	2197 (1028to3989)	1.24 (0.57to2.27)	7347 (3055to13133)	1.13 (0.49to1.98)	-0.28 (-0.55 to -0.01)
High-income North America	4667 (2251to6961)	1.29 (0.62to1.88)	28244 (12799to45533)	4.05 (1.86to6.4)	3.77 (3.52 to 4.03)
North Africa and Middle East	4419 (1942to6922)	3.12 (1.31to5)	16440 (7457to25621)	4.17 (1.85to6.63)	0.97 (0.77 to 1.17)
Oceania	74 (30to136)	2.98 (1.18to5.7)	287 (113to506)	4.57 (1.76to8.27)	1.38 (1.27 to 1.49)
South Asia	2914 (1216to5660)	0.56 (0.24to1.13)	15754 (6786to28269)	1.12 (0.48to2.03)	2.28 (1.95 to 2.6)
Southeast Asia	1833 (646to3904)	0.79 (0.28to1.68)	10263 (3643to22044)	1.67 (0.59to3.62)	2.47 (2.36 to 2.58)
Southern Latin America	1313 (603to1978)	3 (1.37to4.52)	2551 (1173to3911)	2.81 (1.29to4.26)	-0.21 (-0.77 to 0.35)
Southern Sub-Saharan Africa	245 (95to402)	1.02 (0.38to1.74)	936 (365to1567)	1.89 (0.73to3.34)	2.04 (1.74 to 2.33)
Tropical Latin America	2090 (873to3375)	2.63 (1.09to4.4)	9399 (4315to14026)	3.77 (1.72to5.68)	1.23 (0.71 to 1.74)
Western Europe	4453 (2041to6873)	0.74 (0.34to1.13)	11387 (4914to18310)	0.91 (0.39to1.41)	0.7 (0.47 to 0.93)

Western Sub-Saharan Africa	1086 (388to1971)	1.46 (0.52to2.79)	3141 (1377to5301)	1.97 (0.82to3.47)	0.98 (0.85 to 1.12)

Table S2 Cases, ASR and AAPC of death of HBMI-T2DKD by different GBD regions from 1990 to 2021.

ASR, age-standardized rate; AAPC, average annual percent change; DALYs, the Disability-Adjusted Life Years; HBMI-T2DKD, chronic kidney disease due to diabetes mellitus type 2 attributable to high body mass index; ASMR, age-standardized mortality rate.

GBD regions	1990		2021		AAPC
	Cases (95% UI)	ASDR (95% UI)	Cases (95% UI)	ASDR (95% UI)	(95% CI)
Andean Latin America	19405 (7651to29693)	96.96 (37.64to151.31)	86513 (38663to133617)	147.4 (65.4to228.82)	1.48 (1.09 to 1.87)
Australasia	2803 (1462to3827)	12.15 (6.35to16.47)	8665 (4701to11963)	15.98 (8.7to21.88)	0.83 (0.43 to 1.23)
Caribbean	15970 (7014to25427)	62.07 (27.05to99.53)	60834 (26524to93605)	112.51 (49.26to172.98)	1.96 (1.5 to 2.41)
Central Asia	13048 (6594to17962)	27.74 (13.97to38.46)	33078 (18149to45984)	39.17 (21.27to55.11)	1.08 (0.42 to 1.74)
Central Europe	32716 (17770to44575)	21.97 (11.97to29.66)	43859 (22920to63967)	19.72 (10.44to28.06)	-0.38 (-0.65 to -0.11)
Central Latin America	53959 (25535to83738)	65.91 (30.45to104.61)	318450 (159394to480567)	125.48 (62.43to190.67)	2.1 (1.6 to 2.61)
Central Sub-Saharan Africa	11116 (4463to21206)	49.23 (19.26to94.05)	38651 (15190to69360)	70.05 (26.82to129.83)	1.14 (1.02 to 1.27)
East Asia	261721 (93741to564170)	31.37 (11.36to66.67)	993095 (395037to1787618)	45.95 (18.54to82.57)	1.19 (0.93 to 1.46)
Eastern Europe	38076 (19365to52454)	13.74 (6.86to18.81)	65939 (36890to87680)	18.41 (10.32to24.6)	0.94 (0.45 to 1.42)
Eastern Sub-Saharan Africa	24353 (8486to51559)	32.76 (11.45to70.01)	82433 (31225to148717)	49.93 (18.52to92.38)	1.35 (1.25 to 1.46)
High-income Asia Pacific	53801 (25195to92291)	27.53 (12.7to47.96)	127359 (55706to222942)	25.17 (11.56to43.11)	-0.34 (-0.57 to -0.1)
High-income North America	133408 (66239to185639)	38.61 (19.45to53.55)	607345 (285454to911077)	94.03 (44.88to138.06)	2.89 (2.69 to 3.08)
North Africa and Middle East	119890 (56801to179674)	72.24 (33.46to109)	423076 (210977to631071)	93.06 (44.34to140.97)	0.82 (0.65 to 0.99)
Oceania	2439 (998to4377)	77.67 (31.42to141.79)	8561 (3387to15057)	109.87 (43.34to192.6)	1.12 (1 to 1.24)
South Asia	98184 (39673to186530)	16.29 (6.6to31.86)	496214 (217181to861080)	32.31 (14.05to56.5)	2.29 (2.08 to 2.5)
Southeast Asia	58753 (20349to126543)	21.99 (7.69to47.03)	306887 (110217to640064)	44.54 (15.93to93.97)	2.3 (2.2 to 2.4)
Southern Latin America	30034 (14170to43185)	65.49 (30.74to93.96)	51699 (25085to74471)	58.73 (28.84to84.13)	-0.4 (-0.77 to -0.03)
Southern Sub-Saharan Africa	9270 (4178to13819)	33.62 (14.58to50.72)	30524 (14275to48108)	52.65 (23.54to84.77)	1.44 (1.1 to 1.78)
Tropical Latin America	60216 (26267to89549)	66.26 (28.34to100.9)	219886 (104654to313390)	85.51 (40.51to122.33)	0.86 (0.58 to 1.13)
Western Europe	125066 (56308to182818)	21.34 (9.73to30.8)	220490 (99501to327778)	21.57 (9.99to31.71)	0.04 (-0.08 to 0.15)

Western Sub-Saharan Africa	33744 (13091to57151)	38.94 (14.72to67.27)	99519 (48176to152321)	50.51 (23.27to80.56)	0.84 (0.77 to 0.92)

Table S3 Cases, ASR and AAPC of DALYs of HBMI-T2DKD by different GBD regions in 1990-2021.

ASR, age-standardized rate; AAPC, average annual percent change; DALYs, the Disability-Adjusted Life Years; HBMI-T2DKD, chronic kidney disease due to diabetes mellitus type 2 attributable to high body mass index; ASDR, age-standardized DALYs rate.

Nations	Death					DALYs				
	1990		2021		AAPC	1990		2021		AAPC
	Cases (95% UI)	ASMR (95% UI)	Cases (95% UI)	ASMR (95% UI)	(95% CI)	Cases (95% UI)	ASMR (95% UI)	Cases (95% UI)	ASMR (95% UI)	(95% CI)
Afghanistan	228 (87to428)	3.27 (1.24to6.21)	330 (111to673)	3.71 (1.2to8.23)	0.43 (0.34 to 0.51)	6821 (2605to12488)	91.32 (34.81to165.93)	10287 (3761to20111)	96.81 (34.3to202.99)	0.18 (0.08 to 0.29)
Albania	15 (8to23)	0.88 (0.47to1.35)	34 (18to54)	0.8 (0.43to1.27)	-0.41 (-0.85 to 0.04)	506 (295to692)	25.8 (14.77to35.58)	1010 (581to1412)	23.01 (13.36to31.66)	-0.44 (-0.71 to -0.16)
Algeria	198 (82to366)	2.15 (0.84to4.1)	1034 (437to1811)	3.64 (1.55to6.57)	1.75 (1.57 to 1.93)	5608 (2547to9834)	48.12 (21.54to84.24)	24683 (10983to40719)	72.52 (31.72to121.87)	1.31 (1.14 to 1.48)
American Samoa	2 (1to3)	9.27 (3.71to16.8)	11 (5to18)	26 (11.06to43.91)	3.55 (3.21 to 3.88)	52 (24to88)	222.72 (96.95to384.14)	271 (125to422)	565.86 (257.8to891.85)	3.2 (2.88 to 3.52)
Andorra	1 (0to1)	1.25 (0.55to2.11)	2 (1to3)	0.89 (0.36to1.57)	-1.16 (-1.6 to -0.72)	16 (7to24)	29.75 (13.81to45.32)	38 (17to59)	23.25 (10.86to36.08)	-0.84 (-1.09 to -0.58)
Angola	31 (9to74)	0.91 (0.28to2.15)	145 (48to314)	1.45 (0.48to3.1)	1.55 (1.4 to 1.7)	1078 (357to2387)	25.68 (8.32to59.35)	4967 (1700to9772)	38.88 (13.36to79.32)	1.35 (1.19 to 1.5)
Antigua and Barbuda	2 (1to4)	3.96 (1.57to7.62)	9 (4to15)	8.68 (3.45to15.71)	2.61 (1.51 to 3.72)	49 (20to88)	97 (39.47to168.31)	204 (87to330)	188.04 (79.52to310.62)	2.04 (1.01 to 3.09)
Argentina	1092 (500to1658)	3.55 (1.61to5.38)	1901 (868to2845)	3.28 (1.51to4.85)	-0.24 (-0.9 to 0.43)	24472 (11488to35456)	76.21 (35.51to109.48)	38614 (18832to55950)	68.54 (33.65to98.82)	-0.28 (-0.63 to 0.07)
Armenia	2 (1to3)	0.07 (0.03to0.11)	39 (19to60)	0.9 (0.43to1.38)	8.35 (5.78 to 10.99)	483 (211to744)	17.69 (7.93to27.08)	1563 (831to2228)	35.96 (19.29to51.17)	2.28 (1.2 to 3.38)
Australia	50 (25to73)	0.28 (0.14to0.41)	255 (126to378)	0.48 (0.24to0.71)	1.7 (0.91 to 2.5)	2123 (1125to2946)	11.04 (5.8to15.26)	6785 (3674to9484)	14.68 (8to20.34)	0.84 (0.57 to 1.1)
Austria	93 (43to139)	0.75 (0.34to1.08)	444 (216to616)	1.86 (0.92to2.58)	3.05 (2.24 to 3.87)	2743 (1222to3918)	22.67 (10.26to32.17)	7522 (3712to9918)	36.65 (18to48.06)	1.65 (1.11 to 2.18)
Azerbaijan	26 (12to40)	0.53 (0.25to0.82)	88 (45to140)	0.92 (0.46to1.47)	1.78 (1.51 to 2.06)	1599 (814to2247)	31.55 (15.67to44.43)	4347 (2402to6131)	40.4 (22.01to58.12)	0.81 (0.68 to 0.93)
Bahamas	6 (3to10)	4.31 (1.83to6.92)	30 (14to49)	7.71 (3.46to13.06)	1.88 (0.97 to 2.8)	178 (82to262)	112.22 (51.33to167.97)	773 (373to1200)	182.87 (88.23to288.07)	1.61 (0.81 to 2.41)
Bahrain	4 (2to7)	3 (1.26to5.77)	32 (16to52)	5.97 (2.84to10.59)	2.19 (1.61 to 2.76)	120 (59to199)	67.57 (30.88to118.02)	887 (462to1361)	112.68 (56.32to186.4)	1.65 (1.22 to 2.08)
Bangladesh	188 (66to385)	0.43 (0.15to0.89)	853 (325to1692)	0.66 (0.24to1.33)	1.52 (1.23 to 1.82)	6077 (2054to12772)	12.55 (4.27to26.32)	27642 (10931to53588)	19.51 (7.62to37.78)	1.51 (1.4 to 1.63)
Barbados	10 (4to18)	3.61 (1.56to6.07)	36 (17to62)	6.92 (3.2to11.81)	2.24 (1.13 to 3.37)	238 (107to389)	87.83 (40.36to132.71)	767 (357to1243)	148.11 (70.17to238.01)	1.83 (0.82 to 2.84)
Belarus	5 (2to8)	0.04 (0.02to0.07)	29 (14to43)	0.17 (0.08to0.26)	4.42 (3.44 to 5.41)	1328 (588to2113)	10.29 (4.56to16.27)	2412 (1276to3513)	14.86 (7.92to21.63)	1.15 (0.94 to 1.37)
Belgium	132 (59to205)	0.82 (0.36to1.26)	273 (118to443)	0.87 (0.38to1.39)	0.34 (-0.17 to 0.85)	3827 (1715to5803)	24.56 (11.07to36.83)	6145 (2737to8923)	24.79 (11.47to35.18)	-0.03 (-0.41 to 0.35)
Belize	4 (2to7)	4.56 (1.71to7.39)	27 (11to43)	9.75 (3.94to15.66)	2.41 (1.99 to 2.85)	103 (41to153)	110.92 (43.43to165.59)	692 (320to1018)	225.14 (98.4to344.81)	2.24 (1.6 to 2.88)
Benin	24 (8to44)	1.29 (0.42to2.51)	73 (26to139)	1.59 (0.55to3.13)	0.71 (0.52 to 0.9)	696 (235to1200)	34.91 (11.6to61.66)	2333 (852to4008)	42.96 (15.34to77.94)	0.68 (0.45 to 0.91)
Bermuda	2 (1to4)	4.13 (1.9to5.99)	7 (3to10)	4.43 (2.13to6.69)	0.12 (-0.44 to 0.68)	58 (29to80)	94.09 (45.63to128.99)	128 (65to183)	93.58 (48.99to132.89)	-0.12 (-0.61 to 0.38)

Bhutan	4 (1to7)	1.58 (0.59to3.04)	11 (4to21)	1.86 (0.7to3.67)	0.55 (0.4 to 0.69)	119 (45to218)	45.72 (17.28to86.15)	315 (127to581)	51.04 (20.6to94.8)	0.37 (0.24 to 0.49)
Bolivia (Plurinational State of)	152 (54to278)	5.33 (1.86to10.04)	735 (273to1325)	8.84 (3.19to16.48)	1.62 (1.41 to 1.83)	3968 (1424to6854)	124.11 (43.91to218.8)	17481 (6639to29440)	192.02 (71.95to329.58)	1.39 (1.2 to 1.59)
Bosnia and Herzegovina	31 (15to48)	0.87 (0.42to1.37)	59 (29to103)	0.91 (0.44to1.57)	0.23 (-0.23 to 0.69)	1039 (522to1550)	25.91 (12.86to38.86)	1543 (754to2540)	24.36 (12.02to39.42)	-0.17 (-0.52 to 0.18)
Botswana	3 (1to6)	0.64 (0.2to1.26)	17 (6to31)	1.41 (0.46to2.63)	2.58 (1.82 to 3.35)	129 (49to219)	22.27 (8.3to38.52)	601 (242to1012)	40.45 (15.46to68.69)	1.88 (1.34 to 2.43)
Brazil	2026 (843to3298)	2.62 (1.08to4.41)	9136 (4194to13577)	3.74 (1.71to5.6)	1.22 (0.7 to 1.73)	58603 (25499to87666)	66.03 (28.15to101.03)	213637 (101484to304756)	85 (40.2to122)	0.84 (0.55 to 1.12)
Brunei Darussalam	2 (1to4)	2.64 (1.1to5.14)	10 (4to18)	3.65 (1.5to6.74)	0.98 (0.72 to 1.24)	61 (23to110)	58.59 (23.68to109.53)	290 (125to495)	82.33 (35.94to143.18)	1.06 (0.9 to 1.22)
Bulgaria	50 (26to74)	0.49 (0.26to0.71)	153 (74to258)	1.03 (0.5to1.71)	2.35 (1.49 to 3.22)	2238 (1230to3147)	18.63 (10.55to26.13)	4215 (2122to6587)	30.21 (15.53to46.03)	1.5 (0.62 to 2.37)
Burkina Faso	28 (9to66)	0.73 (0.23to1.76)	64 (19to129)	0.78 (0.24to1.63)	0.2 (0 to 0.39)	882 (289to1943)	19.94 (6.4to45.46)	2142 (715to4174)	21.67 (7.1to42.31)	0.29 (0.05 to 0.52)
Burundi	21 (7to47)	1.03 (0.36to2.36)	50 (17to110)	1.31 (0.44to2.83)	0.77 (0.57 to 0.96)	533 (179to1220)	23.44 (8.07to53.37)	1329 (438to2893)	28.36 (9.64to60.92)	0.6 (0.41 to 0.8)
Cabo Verde	2 (1to3)	0.68 (0.24to1.32)	7 (3to13)	1.55 (0.58to3.12)	2.72 (2.17 to 3.28)	49 (19to84)	22.18 (8.62to37.54)	181 (79to312)	39.4 (16.89to69.51)	1.86 (1.45 to 2.27)
Cambodia	19 (6to45)	0.49 (0.16to1.14)	62 (18to155)	0.55 (0.17to1.35)	0.35 (0.28 to 0.42)	590 (169to1441)	13.02 (3.86to30.65)	1929 (548to4817)	14.84 (4.44to37.06)	0.43 (0.35 to 0.51)
Cameroon	152 (48to252)	4.13 (1.3to7.06)	557 (247to956)	5.54 (2.35to9.7)	0.86 (0.55 to 1.17)	4242 (1445to6728)	97.63 (32.58to156.03)	16213 (7814to25694)	130.58 (60.11to213.78)	0.91 (0.71 to 1.11)
Canada	313 (123to498)	0.97 (0.38to1.52)	900 (317to1499)	1.11 (0.4to1.84)	0.43 (-0.32 to 1.2)	7571 (2847to11242)	23.21 (8.78to34.05)	19150 (7130to30294)	26.72 (10.4to41.11)	0.41 (0.02 to 0.8)
Central African Republic	17 (6to35)	1.68 (0.59to3.54)	42 (16to82)	2.21 (0.79to4.4)	0.89 (0.81 to 0.97)	556 (222to1135)	45.5 (17.04to90.69)	1456 (582to2674)	59.14 (22.6to111.53)	0.86 (0.77 to 0.96)
Chad	27 (9to57)	1.07 (0.37to2.19)	58 (20to124)	1.16 (0.39to2.45)	0.28 (0.08 to 0.48)	818 (287to1574)	29.3 (10.23to56.79)	1918 (650to3788)	31.67 (10.92to63.46)	0.27 (0.09 to 0.45)
Chile	165 (77to243)	1.81 (0.84to2.66)	544 (245to904)	2.06 (0.93to3.42)	0.29 (-0.53 to 1.12)	4258 (2104to5986)	43.28 (21.27to61.04)	11052 (5346to17076)	42.56 (20.84to65.44)	-0.16 (-0.78 to 0.47)
China	8208 (2931to17659)	1.26 (0.46to2.64)	37355 (15399to68643)	1.94 (0.79to3.54)	1.25 (0.65 to 1.86)	245323 (86681to531695)	30.29 (10.87to64.73)	941988 (377217to1686112)	45.03 (18.24to80.62)	1.24 (0.96 to 1.52)
Colombia	252 (98to432)	1.64 (0.63to2.91)	936 (380to1592)	1.67 (0.68to2.85)	-0.14 (-0.8 to 0.52)	7625 (3167to12417)	43.68 (17.32to72.09)	24546 (11242to38780)	44.18 (20.18to70.02)	-0.03 (-0.69 to 0.64)
Comoros	2 (1to4)	1.18 (0.4to2.6)	10 (4to23)	2.44 (0.89to5.46)	2.37 (2.23 to 2.5)	51 (16to113)	26.43 (8.54to58.6)	253 (88to546)	52.85 (18.62to113.57)	2.23 (2.07 to 2.4)
Congo	23 (7to45)	2.31 (0.73to4.76)	72 (28to133)	3.07 (1.11to6.2)	0.94 (0.63 to 1.24)	718 (248to1388)	63.1 (21.45to125.87)	2322 (958to3939)	78.49 (31.67to140.97)	0.7 (0.4 to 1)

Cook Islands	1 (0to1)	4.77 (2to8.23)	2 (1to3)	7.26 (3.15to12.01)	1.38 (1.21 to 1.55)	15 (7to24)	119.63 (53.42to196.24)	42 (20to67)	164.95 (77.66to261.19)	1.06 (0.89 to 1.23)
Costa Rica	32 (14to50)	1.92 (0.82to3.1)	202 (87to332)	3.63 (1.57to6.04)	2.08 (1.3 to 2.87)	963 (453to1409)	55.58 (25.64to81.74)	5050 (2408to7835)	90.87 (43.38to140.75)	1.56 (0.85 to 2.27)
Croatia	36 (18to54)	0.67 (0.32to1.01)	96 (44to162)	0.96 (0.44to1.59)	0.35 (0.2 to 0.5)	1274 (702to1777)	21.33 (11.69to30.1)	2221 (1105to3381)	24.12 (12.31to35.61)	0.34 (0.19 to 0.49)
Cuba	111 (45to195)	1.12 (0.45to1.96)	636 (266to1124)	3.15 (1.35to5.47)	1.04 (-0.09 to 2.19)	3317 (1461to5370)	32.73 (14.4to52.87)	14614 (6576to22973)	75.42 (34.7to116.84)	0.35 (-0.76 to 1.48)
Cyprus	9 (3to17)	1.88 (0.76to3.79)	23 (10to45)	1.55 (0.64to2.74)	3.51 (2.87 to 4.15)	208 (86to374)	32.81 (13.57to60.39)	526 (228to926)	28.8 (12.63to46.91)	2.99 (2.32 to 3.67)
Czechia	87 (45to130)	0.62 (0.32to0.91)	106 (49to179)	0.45 (0.21to0.75)	-0.63 (-1.19 to -0.06)	3048 (1723to4143)	22.05 (12.6to29.72)	3264 (1624to5132)	15.1 (7.62to23.11)	-0.42 (-0.75 to -0.09)
Cote-D'Ivoire	48 (18to88)	1.45 (0.5to2.82)	155 (63to271)	1.62 (0.61to2.99)	-1 (-1.61 to -0.38)	1679 (696to2870)	39.83 (15.14to69.93)	5390 (2346to8675)	44.36 (18.52to75.22)	-1.25 (-1.69 to -0.82)
Democratic People's Republic of Korea	186 (66to388)	1.53 (0.55to3.2)	633 (229to1300)	2.15 (0.79to4.45)	1.12 (1.03 to 1.2)	5170 (1810to10793)	35.07 (12.81to73.13)	15570 (5529to31838)	48.86 (17.56to99.84)	1.08 (1.02 to 1.15)
Democratic Republic of the Congo	273 (105to532)	2.14 (0.8to4.1)	936 (336to1838)	3.22 (1.14to6.35)	1.31 (1.22 to 1.39)	8129 (3393to15878)	52.42 (21.44to99.01)	27922 (10820to51233)	76.79 (28.39to145.42)	1.24 (1.09 to 1.39)
Denmark	42 (19to63)	0.47 (0.22to0.7)	181 (84to264)	1.27 (0.59to1.86)	3.36 (2.24 to 4.49)	1456 (627to2128)	17.43 (7.55to25.79)	3612 (1762to5147)	28.32 (13.89to39.57)	1.66 (0.89 to 2.43)
Djibouti	1 (0to2)	0.81 (0.29to1.86)	9 (3to19)	1.83 (0.63to4.13)	2.65 (2.56 to 2.74)	23 (7to55)	17.8 (5.98to40.47)	249 (84to520)	39.48 (13.82to85.95)	2.59 (2.48 to 2.71)
Dominica	4 (1to6)	6.13 (2.49to10.27)	10 (4to16)	12.22 (5.07to20.12)	2.27 (2.16 to 2.38)	80 (33to127)	138.51 (57.71to215.93)	222 (96to350)	263.06 (113.9to413.46)	2.12 (2.03 to 2.2)
Dominican Republic	88 (34to147)	2.74 (1.03to4.59)	482 (205to791)	4.93 (2.09to8.15)	2.08 (1.59 to 2.58)	2427 (912to3849)	66.12 (24.92to106.95)	12099 (5335to18702)	120.25 (52.49to186.59)	2.08 (1.51 to 2.66)
Ecuador	195 (85to309)	4.13 (1.76to6.59)	1251 (564to2207)	8.11 (3.65to13.98)	2.13 (1.42 to 2.85)	4671 (2180to6798)	90.54 (41.43to133.47)	27526 (13189to47887)	170.58 (81.53to295.21)	2.01 (1.19 to 2.83)
Egypt	1108 (454to1854)	5.69 (2.3to9.74)	3914 (1832to6272)	7.9 (3.48to12.91)	1.09 (0.6 to 1.58)	28324 (12159to45576)	116.05 (48.7to187.31)	101914 (50056to158656)	167.61 (78.75to260.9)	1.17 (0.7 to 1.64)
El Salvador	87 (35to143)	3.04 (1.2to5.06)	524 (214to876)	8.26 (3.39to13.76)	3.29 (2.41 to 4.18)	2336 (1001to3639)	79.05 (33.55to123.3)	12135 (5268to19577)	198.75 (86.38to321.3)	3.19 (2.94 to 3.45)
Equatorial Guinea	4 (2to8)	2.45 (0.91to4.65)	18 (7to36)	4.76 (1.72to9.13)	2.18 (1.88 to 2.49)	133 (52to238)	65.92 (25.28to117.27)	512 (215to923)	104.71 (42.43to194.37)	1.57 (1.36 to 1.78)
Eritrea	5 (1to12)	0.54 (0.17to1.29)	25 (8to60)	1.16 (0.37to2.69)	2.52 (2.43 to 2.61)	146 (46to365)	12.85 (4.07to31.31)	728 (211to1793)	26.36 (8.03to63.48)	2.32 (2.21 to 2.44)
Estonia	6 (3to9)	0.31 (0.14to0.46)	54 (25to79)	1.58 (0.77to2.27)	5.42 (4.04 to 6.81)	369 (172to533)	17.91 (8.47to25.79)	1172 (607to1611)	39.66 (21.1to53.1)	2.64 (2.17 to 3.11)
Eswatini	5 (2to9)	2.14 (0.79to3.73)	19 (7to36)	4.13 (1.55to7.86)	2.21 (1.83 to 2.6)	162 (67to265)	57.14 (22.95to93.48)	591 (266to974)	104.8 (45.24to179.22)	2.01 (1.77 to 2.24)
Ethiopia	357 (119to829)	2.16 (0.73to4.97)	759 (250to1653)	2.08 (0.68to4.59)	-0.11 (-0.29 to 0.07)	9582 (3034to22624)	48.59 (16.17to113.83)	18229 (6096to38943)	43.9 (14.49to95.23)	-0.33 (-0.5 to -0.15)

Fiji	19 (8to36)	6.4 (2.62to11.88)	98 (44to160)	15.97 (7.1to26.33)	3.06 (2.81 to 3.31)	598 (253to1049)	158.83 (66.96to286.58)	2551 (1150to4096)	340.7 (152.03to544.57)	2.51 (2.27 to 2.76)
Finland	31 (15to48)	0.42 (0.21to0.66)	103 (45to175)	0.6 (0.27to1.02)	1.18 (0.87 to 1.5)	1074 (538to1621)	14.85 (7.48to22.41)	2120 (991to3350)	15.35 (7.23to23.61)	0.07 (-0.26 to 0.4)
France	660 (277to1054)	0.73 (0.31to1.15)	1580 (729to2254)	0.76 (0.36to1.06)	0.13 (-0.38 to 0.65)	14912 (6686to21812)	17.34 (7.85to25.31)	29780 (14477to41038)	18.47 (9.07to25.52)	0.26 (0.02 to 0.51)
Gabon	18 (8to32)	3.6 (1.45to6.23)	57 (20to103)	7 (2.3to12.81)	2.22 (1.98 to 2.46)	502 (214to824)	88.68 (37.84to145.02)	1473 (571to2565)	147.32 (54.1to261.31)	1.69 (1.46 to 1.92)
Gambia	5 (1to9)	1.55 (0.5to3.15)	18 (7to33)	2.07 (0.73to4.01)	1.01 (0.43 to 1.6)	146 (53to251)	41.26 (14.86to74.13)	540 (225to904)	53.97 (21.39to93.69)	0.94 (0.19 to 1.7)
Georgia	15 (7to25)	0.25 (0.12to0.39)	54 (26to94)	0.89 (0.43to1.53)	4.15 (2.32 to 6.02)	1506 (727to2103)	24.04 (11.33to33.75)	2082 (1044to3252)	36.12 (18to55.64)	1.09 (0.53 to 1.66)
Germany	977 (405to1585)	0.72 (0.3to1.15)	2816 (1081to5269)	1.1 (0.44to2.02)	1.5 (1.09 to 1.91)	28927 (12367to43835)	22.53 (9.77to33.86)	50257 (21402to87061)	23.49 (9.95to39.34)	0.16 (-0.14 to 0.45)
Ghana	71 (24to146)	1.42 (0.51to2.99)	494 (212to872)	3.75 (1.63to6.78)	3.16 (3.05 to 3.27)	1972 (657to3883)	32.49 (11.1to65.16)	12851 (5636to21527)	79.45 (34.85to136.21)	2.93 (2.77 to 3.09)
Greece	358 (172to546)	2.47 (1.16to3.78)	877 (397to1277)	2.69 (1.27to3.82)	0.1 (-1.67 to 1.9)	7389 (3701to10935)	48.79 (24.15to70.77)	14629 (6955to20173)	54.52 (26.98to73.57)	0.43 (-0.65 to 1.51)
Greenland	0 (0to1)	1.57 (0.5to2.68)	1 (0to2)	1.37 (0.4to2.83)	-0.34 (-0.54 to -0.14)	11 (4to18)	36.21 (11.87to60.52)	23 (8to44)	33.99 (10.54to64.1)	-0.13 (-0.28 to 0.02)
Grenada	3 (1to5)	3.95 (1.58to7.31)	10 (4to19)	9.53 (3.73to17.51)	3.03 (2.26 to 3.82)	69 (28to123)	104.79 (42.01to182.24)	262 (112to434)	222.25 (93.79to376.77)	2.58 (2.05 to 3.12)
Guam	3 (1to4)	4.75 (1.94to7.96)	11 (4to19)	4.92 (1.9to8.61)	-0.04 (-1.16 to 1.1)	81 (36to126)	108.72 (48.08to173.46)	300 (119to503)	141.75 (56.72to236.46)	0.76 (0.05 to 1.48)
Guatemala	94 (36to152)	3.51 (1.31to5.58)	492 (205to871)	4.78 (1.99to8.49)	1.05 (0.13 to 1.97)	2638 (1099to3993)	79.89 (32.11to122.05)	13138 (5795to22140)	118.69 (51.64to200.13)	1.3 (0.51 to 2.09)
Guinea	29 (10to56)	0.97 (0.34to1.85)	52 (18to102)	1.04 (0.37to2.08)	0.26 (0.13 to 0.39)	943 (345to1646)	28.27 (10.4to49.94)	1743 (679to3100)	30.04 (11.27to55.7)	0.22 (0.12 to 0.31)
Guinea-Bissau	6 (2to13)	1.73 (0.55to3.68)	11 (4to21)	1.75 (0.6to3.56)	0.02 (-0.1 to 0.14)	192 (64to358)	46.8 (15.56to91.85)	383 (137to690)	47.71 (16.78to89.43)	0.07 (-0.03 to 0.17)
Guyana	15 (7to26)	4.19 (1.84to7.68)	63 (28to107)	10.26 (4.59to17.83)	2.97 (2.33 to 3.62)	417 (198to688)	106.12 (49.65to179.98)	1648 (752to2792)	245.73 (110.85to416.67)	2.92 (1.85 to 3.99)
Haiti	44 (17to95)	1.45 (0.54to3.24)	167 (54to395)	2.43 (0.77to5.73)	1.72 (1.53 to 1.92)	1347 (523to2956)	38.73 (15.01to84.9)	5139 (1757to12024)	64.32 (21.8to147.39)	1.7 (1.49 to 1.91)
Honduras	20 (8to35)	1.08 (0.41to1.88)	137 (50to245)	2.35 (0.84to4.24)	2.6 (2.07 to 3.14)	760 (316to1205)	36.53 (15.01to58.84)	4078 (1599to6979)	63.47 (24.76to110.04)	1.85 (1.33 to 2.37)
Hungary	50 (24to75)	0.34 (0.17to0.51)	122 (52to209)	0.56 (0.24to0.94)	1.74 (1.11 to 2.38)	2209 (1169to3088)	15.08 (7.97to21.07)	3288 (1559to5170)	16.59 (8.2to25.18)	0.37 (0.11 to 0.64)
Iceland	1 (0to1)	0.25 (0.12to0.37)	3 (1to4)	0.37 (0.17to0.56)	1.3 (0.93 to 1.68)	36 (18to49)	12.2 (6.29to16.95)	80 (40to116)	13.54 (6.69to19.46)	0.33 (0.06 to 0.6)
India	1947 (823to3742)	0.46 (0.19to0.91)	11778 (5039to21295)	1.04 (0.45to1.9)	2.67 (2.23 to 3.11)	69074 (28597to130202)	14.02 (5.8to26.71)	371564 (160646to658466)	30.11 (12.91to53.76)	2.53 (2.3 to 2.75)
Indonesia	533 (189to1209)	0.6 (0.21to1.32)	2870 (1086to5787)	1.26 (0.48to2.61)	2.42 (2.33 to 2.52)	18162 (6128to40817)	17.53 (6.15to38.75)	97758 (39117to196286)	36.63 (14.53to73.94)	2.42 (2.34 to 2.5)
Iran (Islamic Republic of)	352 (133to615)	1.67 (0.64to3.08)	1585 (637to2679)	2.31 (0.91to3.98)	1.03 (0.79 to 1.27)	10798 (4189to17660)	41.54 (16.22to70.07)	40567 (17527to63037)	52.84 (21.96to83.54)	0.76 (0.57 to 0.95)
Iraq	364 (176to574)	4.9 (2.35to7.75)	993 (385to1766)	5.14 (1.95to9.24)	0.21 (-0.13 to 0.56)	9315 (4679to14076)	117.4 (58.39to180.31)	25579 (10305to42890)	109.19 (44.32to188.73)	-0.2 (-0.46 to 0.05)
Ireland	31 (14to49)	0.8 (0.36to1.23)	75 (33to118)	0.86 (0.38to1.35)	0.35 (-0.69 to 1.39)	1143 (530to1699)	27.82 (12.89to40.77)	2243 (1018to3302)	27.63 (12.78to40.55)	0.03 (-0.33 to 0.4)

Israel	115 (50to183)	2.57 (1.1to4.03)	257 (108to457)	1.8 (0.77to3.23)	-1.26 (-1.98 to -0.54)	2474 (1137to3745)	51.9 (23.53to77.69)	4797 (2032to8049)	37.05 (15.53to60.99)	-1.13 (-1.96 to -0.3)
Italy	623 (274to983)	0.7 (0.31to1.11)	1510 (627to2556)	0.73 (0.3to1.22)	0.17 (-0.11 to 0.45)	18168 (8312to27406)	20.22 (9.27to30)	28231 (12717to41969)	17.24 (7.92to24.75)	-0.54 (-0.7 to -0.38)
Jamaica	42 (14to88)	2.28 (0.76to4.76)	150 (53to274)	4.72 (1.67to8.51)	2.81 (0.19 to 5.5)	945 (309to1904)	53.69 (17.32to107.56)	3668 (1399to6317)	117.94 (45.12to202.33)	2.93 (0.87 to 5.03)
Japan	1899 (908to3413)	1.22 (0.57to2.2)	6037 (2515to11000)	1.1 (0.49to1.95)	-0.36 (-0.67 to -0.06)	46393 (22425to79364)	27.81 (13.24to48.16)	102513 (46502to180198)	25.97 (12.3to44)	-0.29 (-0.52 to -0.06)
Jordan	57 (25to90)	5.16 (2.23to8.51)	372 (180to589)	6.58 (3.01to10.72)	0.87 (0 to 1.75)	1553 (727to2281)	117.16 (53.03to183.74)	9119 (4700to14083)	129.9 (63.93to204.03)	0.41 (-0.27 to 1.1)
Kazakhstan	51 (25to76)	0.4 (0.2to0.6)	157 (75to261)	0.96 (0.45to1.58)	2.78 (1.14 to 4.44)	3659 (1755to5199)	28.5 (13.73to40.67)	6749 (3351to10043)	37.05 (18.55to55.8)	0.83 (0.18 to 1.49)
Kenya	85 (30to171)	1.2 (0.42to2.53)	617 (229to1088)	3.32 (1.18to6.15)	3.32 (3.13 to 3.5)	2184 (771to4226)	27.19 (9.74to53.45)	16051 (6256to27059)	71.61 (26.95to124.95)	3.17 (3.03 to 3.3)
Kiribati	2 (1to4)	6.29 (2.4to11.18)	7 (3to14)	12.02 (4.59to23.43)	2.15 (2 to 2.29)	66 (27to110)	167.54 (66.85to287.35)	218 (89to388)	288.17 (115.34to534.29)	1.8 (1.64 to 1.95)
Kuwait	15 (7to24)	2.91 (1.17to5.3)	53 (25to83)	2.33 (1.03to3.8)	-0.8 (-2.63 to 1.08)	468 (237to704)	69.77 (31.64to114.01)	1454 (780to2108)	50.17 (25.3to76.88)	-1.1 (-2.66 to 0.48)
Kyrgyzstan	8 (4to12)	0.27 (0.13to0.39)	41 (20to58)	0.88 (0.42to1.24)	3.89 (3.11 to 4.68)	801 (401to1118)	26.61 (13.14to37.59)	2014 (1166to2777)	39.82 (22.2to54.78)	1.25 (0.95 to 1.56)
Lao People's Democratic Republic	25 (8to57)	1.32 (0.46to3.03)	72 (21to172)	1.69 (0.51to4.16)	0.82 (0.75 to 0.88)	737 (235to1699)	34.29 (11.39to78.8)	2169 (621to5275)	43.7 (13.13to103.45)	0.79 (0.74 to 0.84)
Latvia	5 (3to8)	0.15 (0.08to0.22)	33 (17to48)	0.7 (0.36to1.01)	4.98 (4.04 to 5.93)	529 (281to769)	14.71 (7.85to21.24)	1008 (545to1409)	24.14 (13.23to33.66)	1.59 (1.28 to 1.91)
Lebanon	55 (22to96)	2.85 (1.12to4.94)	218 (91to391)	3.38 (1.45to5.92)	0.59 (0.35 to 0.83)	1457 (618to2377)	66.73 (27.71to111.26)	4223 (1949to6797)	69.19 (32.45to110.11)	0.15 (-0.09 to 0.39)
Lesotho	5 (2to9)	0.65 (0.19to1.19)	17 (6to33)	1.77 (0.6to3.6)	3.42 (2.96 to 3.89)	200 (73to312)	23.7 (8.53to37.87)	566 (220to964)	51.66 (19.31to93.02)	2.57 (2.33 to 2.8)
Liberia	24 (10to41)	2.27 (0.91to3.9)	49 (20to82)	2.64 (0.99to4.88)	0.46 (0.25 to 0.67)	708 (312to1120)	60.74 (26.12to97.65)	1561 (709to2404)	68.77 (28.34to111.19)	0.38 (0.15 to 0.62)
Libya	36 (14to61)	2.03 (0.75to3.57)	221 (87to373)	4.76 (1.78to8.42)	2.77 (2.4 to 3.14)	1004 (424to1643)	51.64 (21.14to85.26)	5933 (2508to9466)	109.74 (44.52to180.87)	2.42 (2.15 to 2.69)
Lithuania	5 (2to7)	0.11 (0.05to0.16)	32 (17to46)	0.48 (0.26to0.67)	4.91 (4.05 to 5.77)	612 (295to893)	13.5 (6.5to19.8)	1241 (669to1728)	20.69 (11.24to28.91)	1.42 (1.03 to 1.81)
Luxembourg	6 (3to9)	1.13 (0.56to1.64)	12 (5to22)	0.93 (0.38to1.7)	-0.45 (-0.93 to 0.03)	159 (84to224)	29.32 (15.36to40.66)	252 (114to416)	22.72 (10.46to36.44)	-0.79 (-1.02 to -0.55)
Madagascar	37 (11to90)	0.87 (0.26to2.06)	138 (44to294)	1.67 (0.52to3.59)	2.1 (1.83 to 2.37)	933 (278to2322)	19.21 (5.75to47.39)	3797 (1198to8182)	35.89 (11.72to74.47)	2.03 (1.71 to 2.34)
Malawi	46 (15to105)	1.49 (0.5to3.38)	184 (73to378)	3.02 (1.19to6.3)	2.36 (2.17 to 2.54)	1207 (397to2797)	32.64 (10.62to74.47)	4814 (1811to9510)	66.59 (25.58to134.7)	2.36 (2.19 to 2.54)
Malaysia	198 (84to372)	2.25 (0.94to4.27)	1024 (418to1771)	3.83 (1.56to6.79)	1.79 (1.4 to 2.19)	6143 (2637to10964)	63.95 (27.59to117.18)	28779 (12163to48980)	98.56 (41.09to168.62)	1.48 (1.13 to 1.84)
Maldives	2 (1to5)	2.49 (0.83to5.3)	6 (2to12)	1.87 (0.69to3.97)	-1.08 (-1.29 to -0.87)	73 (23to154)	70.07 (22.74to145.31)	200 (70to372)	51.64 (18.73to99.92)	-1.1 (-1.29 to -0.9)
Mali	52 (20to94)	1.55 (0.6to2.89)	112 (43to225)	1.52 (0.59to3.27)	-0.01 (-0.09 to 0.07)	1625 (680to2847)	40.5 (16.79to70.56)	3493 (1512to6460)	39.23 (16.54to74.76)	-0.08 (-0.17 to 0.01)
Malta	4 (2to7)	1.03 (0.43to1.71)	11 (5to20)	0.96 (0.4to1.67)	-0.29 (-0.74 to 0.17)	109 (50to170)	26.2 (11.92to40.55)	246 (107to406)	24.7 (10.97to38.72)	-0.21 (-0.48 to 0.06)

Marshall Islands	1 (0to2)	6.09 (2.09to13.72)	4 (1to10)	12.02 (2.27to34.79)	2.23 (2.12 to 2.35)	28 (10to56)	159.54 (56.18to339.99)	115 (25to297)	297.63 (63.31to807.74)	2.04 (1.87 to 2.2)
Mauritania	27 (10to45)	3.11 (1.12to5.16)	67 (25to113)	3.74 (1.43to6.34)	0.63 (0.49 to 0.77)	700 (265to1114)	72.43 (27.24to116.08)	1663 (718to2654)	81.24 (33.64to129.88)	0.4 (0.27 to 0.54)
Mauritius	24 (8to48)	3.38 (1.2to6.82)	182 (71to334)	9.93 (3.89to18.24)	3.17 (2.17 to 4.17)	718 (261to1423)	93.02 (33.34to185.81)	4574 (1779to8207)	244 (94.53to435.73)	2.78 (1.75 to 3.82)
Mexico	1193 (543to2159)	3.39 (1.48to6.48)	8244 (3849to12896)	6.76 (3.16to10.61)	2.27 (1.44 to 3.11)	32244 (15878to51663)	78.18 (37.8to130.01)	211516 (106707to322248)	164.45 (81.9to252.71)	2.41 (1.62 to 3.21)
Micronesia (Federated States of)	3 (1to7)	7.54 (2.93to15.11)	10 (4to18)	15.16 (5.24to29.23)	2.28 (2.22 to 2.35)	100 (40to182)	199.27 (79.65to374.2)	301 (118to509)	374.83 (144.11to662.64)	2.06 (2.02 to 2.1)
Monaco	1 (0to1)	0.72 (0.35to1.09)	1 (1to2)	1.11 (0.5to1.71)	1.44 (1.29 to 1.58)	16 (9to23)	22.28 (11.78to31.44)	29 (15to41)	27.48 (14.67to38.41)	0.68 (0.6 to 0.76)
Mongolia	13 (6to20)	1.3 (0.58to1.96)	24 (13to35)	1.22 (0.59to1.82)	-0.25 (-0.7 to 0.2)	532 (260to761)	50.15 (24.15to71.47)	1097 (623to1466)	46.18 (26.07to62.24)	-0.28 (-0.53 to -0.03)
Montenegro	5 (2to8)	0.84 (0.41to1.28)	10 (5to18)	1.15 (0.51to2.01)	1.15 (0.63 to 1.66)	155 (88to219)	24.99 (14.05to35.3)	274 (141to441)	28.39 (14.56to44.59)	0.5 (0.23 to 0.77)
Morocco	226 (96to400)	1.73 (0.71to3.2)	856 (383to1465)	2.74 (1.17to4.74)	1.49 (1.38 to 1.61)	6013 (2886to9558)	42.31 (19.74to69.15)	21299 (10295to35777)	61.63 (29.13to102.01)	1.23 (1.12 to 1.33)
Mozambique	33 (11to74)	0.67 (0.22to1.56)	151 (54to325)	1.63 (0.59to3.62)	2.93 (2.72 to 3.15)	920 (299to2087)	15.56 (5.02to34.99)	4371 (1594to8867)	38.2 (13.94to82.65)	2.95 (2.77 to 3.13)
Myanmar	221 (76to465)	0.97 (0.33to2.1)	526 (184to1124)	1.1 (0.41to2.43)	0.4 (0.34 to 0.46)	7548 (2549to15784)	29.82 (10.05to61.94)	17187 (5900to36324)	32.66 (11.66to69.85)	0.28 (0.18 to 0.37)
Namibia	5 (2to10)	0.92 (0.3to1.83)	19 (7to33)	1.62 (0.58to2.91)	1.9 (1.59 to 2.2)	204 (79to363)	30.47 (11.17to54.29)	640 (273to1027)	46.2 (18.7to75.66)	1.37 (1.17 to 1.57)
Nauru	0 (0to1)	8.8 (3.46to17.08)	1 (0to2)	18.49 (7.27to33.71)	2.45 (2.29 to 2.62)	12 (5to21)	237.12 (95.02to434.34)	28 (13to44)	453.6 (193.22to755.07)	2.11 (2 to 2.21)
Nepal	47 (15to98)	0.52 (0.17to1.06)	254 (98to534)	1.1 (0.43to2.29)	2.45 (2.31 to 2.59)	1887 (647to3745)	18.23 (6.24to36.1)	9218 (3713to18094)	36.95 (14.85to72.6)	2.3 (2.15 to 2.44)
Netherlands	102 (41to164)	0.5 (0.2to0.81)	359 (150to546)	0.87 (0.37to1.32)	1.81 (1.2 to 2.42)	3363 (1451to5168)	16.75 (7.23to25.58)	7888 (3571to11400)	21.53 (10.02to31.21)	0.86 (0.6 to 1.13)
New Zealand	19 (10to28)	0.51 (0.26to0.73)	71 (34to103)	0.79 (0.38to1.14)	1.26 (0.46 to 2.06)	680 (371to930)	17.79 (9.75to24.19)	1880 (1015to2598)	22.67 (12.54to30.99)	0.76 (0.32 to 1.19)
Nicaragua	36 (13to62)	2.54 (0.9to4.43)	267 (114to459)	5.74 (2.42to9.86)	2.7 (2.23 to 3.17)	1121 (456to1763)	71.45 (27.32to115.04)	7524 (3399to12475)	150.41 (67.02to252.71)	2.45 (2.04 to 2.87)
Niger	18 (6to34)	0.75 (0.25to1.55)	45 (16to91)	0.63 (0.21to1.35)	-0.55 (-0.68 to -0.43)	645 (245to1142)	22.49 (8.19to41.69)	1699 (652to3127)	19.58 (7.46to37.58)	-0.43 (-0.53 to -0.34)
Nigeria	481 (162to920)	1.3 (0.43to2.52)	1127 (462to1908)	1.58 (0.62to2.76)	0.65 (0.47 to 0.82)	15763 (5586to27181)	36.55 (12.71to63.77)	40045 (19193to58813)	44.25 (19.86to68.75)	0.62 (0.46 to 0.78)
Niue	0 (0to0)	6.87 (2.65to12.69)	0 (0to1)	17.11 (6.1to33.17)	2.99 (2.88 to 3.1)	4 (1to6)	172.14 (70.61to302.48)	8 (3to15)	375.02 (144.47to694.95)	2.55 (2.41 to 2.68)
North Macedonia	10 (4to16)	0.58 (0.25to0.94)	21 (8to38)	0.72 (0.29to1.31)	0.72 (0.39 to 1.06)	436 (221to637)	23.38 (11.69to34.24)	780 (372to1261)	23.77 (11.37to38.07)	0.07 (-0.25 to 0.39)
Northern Mariana Islands	1 (1to2)	11.45 (4.82to19.88)	9 (4to14)	20.31 (8.26to34.68)	1.66 (1.32 to 1.99)	46 (19to73)	253.03 (106.94to427.37)	229 (96to361)	434.07 (181.92to697.88)	1.69 (1.46 to 1.92)
Norway	19 (9to31)	0.24 (0.11to0.39)	54 (25to92)	0.44 (0.2to0.74)	1.83 (0.81 to 2.86)	860 (414to1290)	12.66 (6.15to18.77)	1456 (689to2135)	14.06 (6.82to20.38)	0.36 (0.1 to 0.62)

Oman	11 (4to21)	1.89 (0.65to3.65)	80 (39to126)	5.28 (2.4to8.63)	3.42 (2.81 to 4.02)	348 (132to595)	48.99 (17.4to85.53)	2261 (1243to3431)	114.83 (57.37to177.65)	2.84 (2.53 to 3.16)
Pakistan	728 (299to1329)	1.39 (0.56to2.57)	2858 (1258to5195)	2.58 (1.12to4.71)	2 (1.82 to 2.17)	21026 (8677to38261)	36.67 (15.02to67.23)	87476 (39292to147325)	67.43 (29.45to116.67)	2 (1.87 to 2.13)
Palau	1 (0to1)	7.75 (3.03to14.12)	3 (1to5)	15.84 (6.61to27.88)	2.3 (2.17 to 2.44)	18 (8to31)	185.94 (77.68to320.51)	76 (34to127)	340.53 (146.78to576.45)	1.97 (1.81 to 2.12)
Palestine	30 (13to52)	4.01 (1.75to6.9)	89 (38to146)	4.46 (1.79to7.58)	0.3 (0.06 to 0.55)	744 (354to1220)	88.12 (41.06to145.33)	2294 (1117to3591)	93.36 (42.14to148.85)	0.16 (-0.09 to 0.41)
Panama	25 (10to37)	1.77 (0.74to2.65)	178 (78to288)	3.95 (1.73to6.44)	2.78 (2.13 to 3.42)	730 (312to1059)	50.03 (21.42to72.59)	4165 (1904to6472)	93.99 (42.86to146.66)	2.16 (1.66 to 2.66)
Papua New Guinea	21 (7to45)	1.21 (0.42to2.53)	72 (23to158)	1.44 (0.47to3.18)	0.59 (0.39 to 0.79)	803 (285to1649)	38.31 (13.67to80.07)	2673 (910to5616)	43.83 (14.62to92.27)	0.41 (0.17 to 0.65)
Paraguay	64 (27to100)	3.15 (1.29to4.92)	263 (112to444)	4.77 (1.98to8.12)	1.47 (1.1 to 1.83)	1613 (684to2352)	74.33 (31.49to111.01)	6248 (2863to9725)	107.73 (48.51to171.94)	1.32 (1.02 to 1.62)
Peru	465 (168to772)	4.25 (1.54to7.16)	1953 (798to3294)	5.89 (2.4to9.99)	1.15 (0.15 to 2.17)	10766 (3968to17114)	92.4 (33.75to150.18)	41507 (17833to65266)	124.47 (53to197.73)	1.06 (0.16 to 1.96)
Philippines	252 (75to565)	1.01 (0.32to2.3)	1928 (562to4351)	2.45 (0.74to5.52)	2.91 (2.65 to 3.18)	8088 (2363to18757)	26.11 (7.74to59.18)	58420 (16772to129459)	65.88 (19.06to147.04)	3.08 (2.91 to 3.25)
Poland	394 (194to586)	0.93 (0.46to1.37)	374 (177to627)	0.48 (0.23to0.8)	-2.15 (-2.83 to -1.46)	11958 (6238to16921)	27.37 (14.39to38.26)	11832 (5842to17894)	16.47 (8.27to24.47)	-1.71 (-2.22 to -1.19)
Portugal	153 (72to247)	1.19 (0.57to1.94)	431 (176to776)	1.31 (0.54to2.33)	0.33 (-0.19 to 0.85)	3916 (1863to6078)	28.7 (13.9to43.93)	7176 (3126to12241)	26.27 (12.02to42.72)	-0.29 (-0.71 to 0.12)
Puerto Rico	223 (102to380)	6.51 (2.94to11.06)	698 (332to1164)	8.52 (4.11to13.5)	0.73 (-0.25 to 1.72)	5047 (2406to7908)	142 (67.24to220.08)	12938 (6442to20162)	182.76 (93.44to275.85)	0.69 (-0.26 to 1.65)
Qatar	2 (1to4)	3.43 (1.31to6.78)	25 (13to39)	5.1 (2.58to8.46)	1.11 (0.36 to 1.87)	86 (43to140)	74.73 (31.63to133.83)	812 (471to1206)	98.02 (52.06to155.67)	0.92 (-0.1 to 1.95)
Republic of Korea	272 (110to518)	1.33 (0.56to2.54)	1178 (450to2344)	1.3 (0.49to2.58)	-0.22 (-0.76 to 0.32)	6712 (2682to12933)	25.58 (10.51to48.11)	21872 (8633to41011)	23.45 (9.21to44.15)	-0.4 (-0.68 to -0.11)
Republic of Moldova	4 (2to6)	0.11 (0.05to0.15)	15 (8to23)	0.25 (0.13to0.38)	2.37 (0.47 to 4.32)	746 (387to1079)	17.28 (8.83to25.04)	1413 (812to2021)	23.51 (13.43to33.35)	0.95 (0.62 to 1.29)
Romania	62 (33to96)	0.25 (0.13to0.38)	181 (104to256)	0.45 (0.26to0.63)	1.96 (0.87 to 3.06)	4081 (2310to5552)	14.84 (8.43to20.33)	7215 (4187to9654)	19.54 (11.5to26.17)	0.79 (0.25 to 1.32)
Russian Federation	512 (242to750)	0.29 (0.14to0.43)	1590 (803to2247)	0.65 (0.33to0.91)	2.36 (0.85 to 3.9)	26680 (13437to36345)	14.91 (7.33to20.21)	46658 (26006to62274)	19.12 (10.66to25.45)	0.79 (0.2 to 1.38)
Rwanda	23 (7to56)	0.99 (0.32to2.32)	65 (22to150)	1.29 (0.42to2.89)	0.85 (0.69 to 1)	629 (199to1536)	22.57 (7.18to53.99)	1674 (568to3968)	27.53 (9.32to64.34)	0.62 (0.36 to 0.87)
Saint Kitts and Nevis	2 (1to4)	6.13 (2.48to11.21)	7 (3to12)	11.56 (4.82to20.4)	2.11 (1.37 to 2.85)	52 (21to93)	148.41 (61.49to247.69)	178 (76to284)	249.84 (106.7to413.39)	1.71 (1.01 to 2.41)
Saint Lucia	3 (1to6)	3.98 (1.64to7.5)	15 (6to28)	6.37 (2.54to12.09)	1.49 (1.1 to 1.88)	83 (34to149)	96.67 (39.55to174.07)	359 (150to616)	147.99 (62.31to253.34)	1.38 (0.61 to 2.15)
Saint Vincent and the Grenadines	2 (1to3)	2.44 (0.95to4.74)	8 (3to15)	5.74 (2.19to10.95)	2.74 (1.87 to 3.62)	42 (16to77)	60.52 (23.31to111.2)	192 (76to339)	134.33 (53.54to235.61)	2.54 (1.7 to 3.37)

Samoa	6 (2to11)	7.47 (2.73to13.65)	18 (8to31)	13.74 (5.63to23.44)	1.99 (1.86 to 2.12)	171 (65to295)	193.64 (72.65to336.66)	475 (212to756)	324.12 (140.46to523.57)	1.68 (1.57 to 1.79)
San Marino	0 (0to0)	0.61 (0.29to0.91)	0 (0to1)	0.42 (0.17to0.73)	-1.26 (-1.81 to -0.71)	7 (4to10)	19.66 (9.76to27.52)	13 (6to20)	17.12 (8.45to25.16)	-0.47 (-0.69 to -0.25)
Sao Tome and Principe	2 (1to3)	3.1 (1.1to5.53)	5 (2to8)	5.31 (2.15to9.06)	1.75 (1.6 to 1.91)	45 (17to72)	71.02 (26.75to113.86)	133 (60to207)	119.45 (51.86to191.96)	1.69 (1.53 to 1.84)
Saudi Arabia	243 (101to416)	4.58 (1.83to8.37)	1767 (865to2773)	11.45 (5.23to18.92)	2.99 (2.83 to 3.14)	7173 (3092to11500)	115.47 (48.71to190.18)	54076 (28024to82083)	253.91 (121.85to407.27)	2.56 (2.38 to 2.74)
Senegal	56 (22to104)	1.93 (0.74to3.76)	142 (55to276)	2.09 (0.77to4.29)	0.21 (-0.17 to 0.6)	1594 (652to2797)	48.67 (19.53to87.73)	3935 (1625to7263)	49.74 (19.86to92.86)	0.05 (-0.36 to 0.45)
Serbia	108 (47to176)	1.17 (0.51to1.92)	197 (85to348)	1.12 (0.48to1.94)	-0.12 (-0.41 to 0.17)	3040 (1467to4616)	28.33 (13.44to43.84)	4540 (2013to7421)	27.06 (12.14to42.85)	-0.06 (-0.41 to 0.28)
Seychelles	2 (1to3)	3.18 (1.34to5.66)	7 (3to14)	6.69 (2.5to12.88)	2.38 (1.82 to 2.94)	51 (23to85)	90.77 (40.39to152.32)	194 (83to338)	161.38 (67.21to285.69)	1.78 (1.48 to 2.09)
Sierra Leone	21 (7to42)	1.15 (0.39to2.35)	39 (14to78)	1.2 (0.43to2.4)	0.14 (0.03 to 0.24)	603 (216to1144)	29.97 (10.56to57.66)	1287 (505to2230)	33.02 (12.45to61.42)	0.31 (0.2 to 0.42)
Singapore	24 (10to44)	1.28 (0.56to2.36)	122 (48to221)	1.48 (0.57to2.68)	0.46 (-0.19 to 1.11)	636 (274to1122)	29.45 (12.69to52.59)	2683 (1160to4698)	31.44 (13.45to55.23)	0.18 (-0.53 to 0.89)
Slovakia	58 (30to83)	0.97 (0.51to1.38)	80 (39to124)	0.82 (0.4to1.26)	-0.55 (-0.9 to -0.2)	1805 (1044to2456)	30.04 (17.22to40.77)	2349 (1284to3285)	24.46 (13.57to33.81)	-0.68 (-0.93 to -0.44)
Slovenia	10 (6to14)	0.41 (0.23to0.56)	24 (11to39)	0.44 (0.21to0.7)	0.34 (-0.16 to 0.83)	403 (223to536)	16.35 (9.1to21.71)	690 (362to995)	15.03 (8.05to21.41)	-0.26 (-0.56 to 0.04)
Solomon Islands	4 (1to10)	3.25 (1.03to7.49)	11 (4to25)	3.45 (1.08to7.89)	0.18 (-0.25 to 0.61)	145 (43to320)	92.94 (28.7to208.61)	395 (116to860)	97.47 (30.64to211.35)	0.13 (-0.36 to 0.62)
Somalia	28 (9to69)	1.43 (0.45to3.49)	110 (36to233)	2.17 (0.7to4.67)	1.38 (1.3 to 1.45)	841 (259to2018)	34.11 (11.08to84.18)	3329 (1088to7172)	52.26 (17.14to107.83)	1.4 (1.29 to 1.51)
South Africa	204 (79to335)	1.09 (0.41to1.83)	767 (311to1280)	1.9 (0.76to3.29)	1.8 (1.32 to 2.28)	7688 (3531to10996)	36.49 (16.09to52.96)	24668 (12008to38324)	53.12 (24.85to84.15)	1.17 (0.75 to 1.58)
South Sudan	25 (8to61)	1.1 (0.38to2.66)	70 (23to152)	2.18 (0.74to4.8)	2.24 (2.15 to 2.33)	608 (206to1517)	24.26 (8.51to59.69)	1910 (650to4182)	48.92 (16.62to105.55)	2.29 (2.18 to 2.4)
Spain	594 (260to1006)	1.12 (0.49to1.87)	1236 (452to2265)	0.88 (0.34to1.55)	-0.77 (-1.01 to -0.53)	14632 (6565to22792)	27.02 (12.23to41.81)	22674 (9790to37447)	20.66 (9.45to32.43)	-0.88 (-1.07 to -0.69)
Sri Lanka	76 (23to172)	0.78 (0.24to1.78)	341 (103to826)	1.32 (0.39to3.22)	1.79 (1.28 to 2.3)	2385 (740to5386)	21.17 (6.66to47.4)	9593 (3045to21892)	35.2 (10.87to80.62)	1.7 (1.34 to 2.07)
Sudan	93 (35to182)	1.07 (0.39to2.03)	310 (114to576)	1.77 (0.62to3.37)	1.59 (1.42 to 1.75)	3017 (1239to5333)	31.36 (12.56to55.06)	9385 (3839to16131)	45.64 (17.61to81.18)	1.21 (1.09 to 1.33)
Suriname	8 (3to15)	3.21 (1.28to6.11)	37 (12to73)	5.91 (1.91to11.65)	2.06 (1.55 to 2.57)	233 (97to412)	86.34 (35.06to155.53)	960 (348to1815)	147.1 (52.15to276.56)	1.79 (1.31 to 2.27)
Sweden	63 (27to102)	0.36 (0.15to0.58)	242 (99to403)	0.83 (0.34to1.38)	2.66 (1.86 to 3.46)	2106 (932to3233)	13.46 (5.97to20.17)	4365 (1975to6655)	18 (8.55to26.8)	0.94 (0.54 to 1.34)
Switzerland	53 (20to99)	0.46 (0.18to0.85)	142 (47to288)	0.56 (0.19to1.11)	0.64 (-0.06 to 1.35)	1718 (692to2875)	16.39 (6.69to27.01)	2986 (1090to5559)	15.36 (6.03to27.2)	-0.17 (-0.64 to 0.29)
Syrian Arab Republic	186 (75to297)	4.04 (1.6to6.69)	610 (273to1016)	5.44 (2.3to9.47)	0.96 (0.62 to 1.3)	4984 (2167to7607)	94.58 (39.65to145.22)	15629 (7660to24387)	117.75 (55.68to188.28)	0.7 (0.43 to 0.98)
Taiwan (Province of China)	460 (180to899)	3.92 (1.52to7.51)	1737 (631to3361)	3.83 (1.4to7.4)	-0.11 (-0.72 to 0.5)	11228 (4428to21329)	78.45 (30.76to149.38)	35538 (13458to64864)	82 (31.16to150.69)	0.12 (-0.39 to 0.63)

Tajikistan	4 (2to6)	0.13 (0.06to0.21)	10 (4to16)	0.18 (0.08to0.31)	1.08 (0.67 to 1.48)	554 (285to817)	20.4 (10.42to30.15)	1286 (665to1928)	21.65 (10.66to32.95)	0.18 (0.08 to 0.29)
Thailand	335 (110to788)	1.06 (0.36to2.42)	2560 (741to5807)	2.33 (0.68to5.26)	2.52 (2.37 to 2.67)	10465 (3443to23795)	28.48 (9.7to66.01)	67888 (21607to150241)	61.99 (19.66to136.66)	2.54 (2.44 to 2.65)
Timor-Leste	1 (0to3)	0.48 (0.16to1.18)	4 (1to10)	0.55 (0.17to1.3)	0.35 (0.16 to 0.54)	31 (10to82)	11.37 (3.67to28.93)	127 (40to309)	14.55 (4.71to35.11)	0.8 (0.65 to 0.95)
Togo	15 (5to29)	1.49 (0.49to2.99)	66 (24to123)	2.29 (0.83to4.36)	1.39 (1.23 to 1.54)	441 (153to780)	37 (12.61to68.8)	2007 (766to3374)	54.29 (20.54to95.19)	1.25 (1.14 to 1.36)
Tokelau	0 (0to0)	4.91 (1.94to9.84)	0 (0to0)	9.67 (3.71to17.23)	2.22 (2.13 to 2.32)	2 (1to3)	124.45 (50.51to239.18)	3 (1to6)	220.92 (87.84to378.24)	1.87 (1.82 to 1.93)
Tonga	2 (1to3)	3.46 (1.21to6.5)	5 (2to9)	7.06 (2.61to11.83)	2.42 (2.24 to 2.59)	61 (23to102)	103.31 (39.68to173.81)	141 (59to220)	175.34 (72.23to273.29)	1.76 (1.63 to 1.9)
Trinidad and Tobago	25 (10to45)	3.29 (1.34to5.94)	155 (66to270)	7.89 (3.36to13.76)	2.93 (2.01 to 3.86)	670 (277to1102)	80.04 (32.88to133.47)	3759 (1678to6236)	189.76 (85.96to314.44)	2.88 (2.08 to 3.68)
Tunisia	58 (22to112)	1.26 (0.49to2.5)	349 (132to638)	2.84 (1.06to5.25)	2.61 (2.42 to 2.81)	1741 (704to3015)	33.71 (13.5to60.95)	8265 (3399to14311)	62.5 (25.39to107.7)	1.98 (1.83 to 2.12)
Turkey	1087 (470to1660)	3.67 (1.52to5.67)	3278 (1472to5293)	3.77 (1.69to6.12)	0.18 (-0.28 to 0.65)	28212 (12905to41333)	83.73 (37.56to123.62)	73908 (35278to111800)	79.48 (38.07to121.21)	-0.1 (-0.43 to 0.22)
Turkmenistan	13 (7to19)	0.7 (0.35to1)	59 (29to95)	1.52 (0.75to2.44)	2.54 (1.34 to 3.76)	736 (378to1007)	37.22 (18.84to51.25)	2429 (1304to3467)	57.15 (30.52to82.38)	1.36 (0.52 to 2.2)
Tuvalu	0 (0to1)	4.24 (1.38to9.04)	1 (0to2)	9.05 (2.59to20)	2.49 (2.41 to 2.57)	8 (3to17)	117.57 (38.23to236.01)	24 (7to48)	221.22 (65.94to451.23)	2.07 (1.99 to 2.14)
Uganda	59 (21to135)	1.04 (0.38to2.4)	248 (97to520)	1.98 (0.77to4.13)	2.06 (1.94 to 2.18)	1607 (559to3613)	24.98 (8.85to56.9)	6786 (2523to13932)	45.6 (17.68to94.99)	1.94 (1.83 to 2.05)
Ukraine	5 (2to7)	0.01 (0to0.01)	106 (50to161)	0.13 (0.06to0.2)	10.05 (8.18 to 11.95)	7813 (4032to11852)	11.04 (5.75to16.61)	12035 (6634to17560)	15.38 (8.49to22.36)	1.03 (0.83 to 1.24)
United Arab Emirates	8 (4to13)	2.12 (0.87to3.94)	105 (48to165)	5.83 (2.18to10.11)	3.29 (1.26 to 5.36)	306 (158to476)	56.51 (24.99to96.26)	3900 (2087to5684)	117.87 (49.63to193.05)	2.42 (0.89 to 3.97)
United Kingdom	384 (160to578)	0.4 (0.17to0.6)	746 (291to1350)	0.47 (0.18to0.83)	0.48 (-0.25 to 1.22)	15706 (6765to23359)	17.43 (7.62to25.76)	23229 (9752to37337)	17.8 (7.64to27.99)	0.03 (-0.25 to 0.31)
United Republic of Tanzania	133 (50to245)	1.42 (0.51to2.63)	454 (191to766)	2.14 (0.87to3.66)	1.36 (1.22 to 1.49)	3685 (1427to6254)	34.2 (13.04to58.8)	11996 (5704to18891)	48.57 (22.01to77.97)	1.14 (1 to 1.28)
United States of America	4353 (2116to6483)	1.32 (0.65to1.93)	27342 (12447to44082)	4.42 (2.04to7)	0.52 (0.13 to 0.91)	125822 (63239to175112)	40.29 (20.46to55.83)	588162 (278742to883858)	102.24 (49.26to150.17)	0.37 (0.01 to 0.73)
United States Virgin Islands	3 (1to4)	3.62 (1.55to6.17)	8 (3to14)	4.34 (1.84to7.53)	3.98 (3.71 to 4.24)	76 (36to115)	87.49 (40.54to138.4)	174 (78to286)	99.46 (46.14to161.98)	3.02 (2.78 to 3.25)
Uruguay	57 (22to81)	1.44 (0.58to2.03)	105 (41to171)	1.63 (0.65to2.63)	0.16 (-0.81 to 1.13)	1303 (517to1850)	33.13 (13.29to46.4)	2030 (866to3104)	36.13 (15.89to54.97)	0.09 (-0.73 to 0.91)
Uzbekistan	47 (19to88)	0.45 (0.17to0.85)	248 (129to382)	1.03 (0.51to1.69)	2.85 (0.71 to 5.04)	3178 (1596to4755)	27.94 (14.09to41.69)	11512 (6214to16534)	41.65 (21.48to61.88)	1.36 (0.35 to 2.38)
Vanuatu	2 (1to5)	4.54 (1.88to9.19)	11 (4to21)	6.86 (2.66to13.6)	1.34 (1.15 to 1.53)	74 (31to149)	113.94 (48.62to228.12)	328 (127to609)	174.51 (67.42to330.77)	1.38 (1.17 to 1.6)

Venezuela (Bolivarian Republic of)	173 (80to257)	1.99 (0.91to2.97)	1440 (632to2358)	4.92 (2.14to8.06)	2.91 (2.46 to 3.36)	5541 (2730to7789)	57.8 (28.05to82.31)	36297 (17047to58459)	118.68 (55.24to190.27)	2.34 (1.98 to 2.7)
Viet Nam	143 (47to327)	0.39 (0.12to0.91)	666 (200to1609)	0.74 (0.24to1.8)	2.07 (1.99 to 2.14)	3678 (1153to8309)	9.27 (2.96to21.14)	17640 (5362to43652)	17.35 (5.26to41.47)	2.06 (2.01 to 2.12)
Yemen	56 (21to115)	1.28 (0.48to2.66)	203 (80to385)	1.64 (0.61to3.2)	0.82 (0.7 to 0.93)	1733 (676to3406)	34.17 (13.35to67.08)	6206 (2668to10720)	42.45 (17.3to77.15)	0.71 (0.59 to 0.84)
Zambia	50 (17to112)	2 (0.65to4.45)	241 (89to467)	4.03 (1.47to7.93)	2.29 (2.14 to 2.44)	1386 (446to3070)	48.06 (15.98to107.51)	6845 (2614to12763)	95.61 (36.4to183.54)	2.24 (2.05 to 2.44)
Zimbabwe	22 (7to49)	0.59 (0.19to1.31)	98 (32to192)	1.62 (0.5to3.29)	3.39 (3 to 3.79)	887 (304to1721)	20.55 (6.96to41.11)	3459 (1321to6023)	47.23 (17.66to84.74)	2.81 (2.49 to 3.12)

Table S4 Cases, ASR and AAPC of death and DALYs of HBMI-T2DKD by different countries/territories in 1990-2021.

ASR, age-standardized rate; AAPC, average annual percent change; DALYs, the Disability-Adjusted Life Years; HBMI-T2DKD, chronic kidney disease due to diabetes mellitus type 2 attributable to high body mass index; ASMR, age-standardized mortality rate; ASDR, age-standardized DALYs rate.

Year	Death		DALYs	
	Cases (95%UI)	ASMR (95%UI)	Cases (95%UI)	ASDR (95%UI)
2022	182809 (174731 to 190886)	2.1 (2.01 to 2.19)	4615841 (4386468 to 4845213)	51.64 (49.07 to 54.2)
2023	190643 (180463 to 200824)	2.13 (2.01 to 2.24)	4811881 (4536400 to 5087361)	52.44 (49.44 to 55.44)
2024	199243 (185765 to 212721)	2.16 (2.01 to 2.3)	5018793 (4671306 to 5366280)	53.24 (49.56 to 56.93)
2025	208282 (190374 to 226189)	2.18 (1.99 to 2.37)	5233363 (4787988 to 5678739)	54.05 (49.45 to 58.65)
2026	217534 (194123 to 240944)	2.21 (1.97 to 2.45)	5452893 (4884669 to 6021117)	54.86 (49.14 to 60.57)
2027	226829 (196874 to 256784)	2.24 (1.94 to 2.53)	5675525 (4960179 to 6390872)	55.67 (48.65 to 62.68)
2028	236590 (199000 to 274179)	2.27 (1.91 to 2.63)	5905392 (5018504 to 6792280)	56.49 (48 to 64.97)
2029	247123 (200710 to 293536)	2.3 (1.86 to 2.73)	6145973 (5062066 to 7229879)	57.32 (47.21 to 67.43)
2030	258211 (201737 to 314685)	2.33 (1.82 to 2.84)	6394815 (5087651 to 7701979)	58.15 (46.26 to 70.04)
2031	269672 (201846 to 337499)	2.36 (1.76 to 2.95)	6649550 (5091928 to 8207172)	58.99 (45.17 to 72.81)
2032	281353 (200830 to 361876)	2.39 (1.7 to 3.07)	6908827 (5072355 to 8745299)	59.83 (43.92 to 75.74)
2033	293645 (198891 to 388399)	2.42 (1.64 to 3.2)	7177121 (5030929 to 9323313)	60.69 (42.53 to 78.84)
2034	306860 (196107 to 417612)	2.45 (1.57 to 3.34)	7458008 (4968156 to 9947859)	61.56 (41 to 82.12)
2035	320812 (192190 to 449434)	2.49 (1.49 to 3.49)	7748951 (4879815 to 10618088)	62.45 (39.32 to 85.58)
2036	335301 (186848 to 483755)	2.53 (1.41 to 3.64)	8046975 (4761393 to 11332557)	63.34 (37.47 to 89.21)
2037	350146 (179812 to 520480)	2.56 (1.32 to 3.81)	8350607 (4609416 to 12091798)	64.24 (35.45 to 93.03)
2038	365803 (171160 to 560447)	2.6 (1.22 to 3.99)	8666076 (4424864 to 12907288)	65.17 (33.27 to 97.08)
2039	382623 (160818 to 604428)	2.64 (1.11 to 4.17)	8997600 (4206391 to 13788809)	66.13 (30.9 to 101.36)
2040	400352 (148392 to 652312)	2.68 (0.99 to 4.37)	9341227 (3947959 to 14734494)	67.11 (28.35 to 105.87)
2041	418699 (133491 to 703908)	2.73 (0.87 to 4.59)	9692292 (3643402 to 15741182)	68.1 (25.58 to 110.62)
2042	437397 (115756 to 759042)	2.77 (0.73 to 4.81)	10048196 (3287693 to 16808698)	69.11 (22.59 to 115.63)
2043	456917 (95048 to 818802)	2.82 (0.59 to 5.05)	10415818 (2879151 to 17952484)	70.16 (19.37 to 120.95)
2044	477709 (71083 to 884372)	2.87 (0.43 to 5.32)	10800744 (2413870 to 19187619)	71.25 (15.89 to 126.61)
2045	499601 (43405 to 955912)	2.92 (0.25 to 5.6)	11199762 (1884614 to 20515036)	72.38 (12.14 to 132.62)
2046	522403 (13640 to 1033562)	2.98 (0.08 to 5.9)	11609102 (1284200 to 21934821)	73.54 (8.1 to 139)
2047	545955 (0 to 1117540)	3.04 (0 to 6.22)	12027584 (606833 to 23451999)	74.73 (3.74 to 145.77)
2048	570814 (0 to 1209569)	3.1 (0 to 6.58)	12464100 (9858 to 25091639)	75.99 (0.06 to 153.04)
2049	597495 (0 to 1311513)	3.17 (0 to 6.96)	12925045 (0 to 26876904)	77.32 (0 to 160.85)

Table S5 Projection of HBMI-T2DKD from 2022 to 2049.

HBMI-T2DKD, chronic kidney disease due to diabetes mellitus type 2 attributable to high body mass index; DALYs, the Disability-Adjusted Life Years; ASMR, age-standardized mortality rate; ASDR, age-standardized DALYs rate.
