# Supplementary material for: Causal impact of elevated body mass index on diabetic kidney disease: an integrated Mendelian randomization and Global Burden of Disease Study 2021 analysis
Source: Ren Fail. 2025 Mar 17;47(1):2472981. doi: 10.1080/0886022X.2025.2472981 (PMC11984565; doi:10.1080/0886022X.2025.2472981)
Supplement: Online Supplementary Material 2.docx [file IRNF_A_2472981_SM0344.docx]

**Online Supplementary Material 2**

**Title:** Causal Impact of Elevated Body Mass Index on Diabetic Kidney Disease: An Integrated Mendelian Randomization and Global Burden of Disease 2021 Analysis

**Authors:** Ye-xin Chen^†^, Dong-sen Hu^†^, Mao-xuan Lin, Zi-heng Gao, Han-zhang Hong, Yu-xin Hu, Ling-zi Yao, Gai-wen Cui, Lin Wang^*^

**FigS1.** SNPs forest plot

**FigS2.** Scatter plot

**FigS3.** Funnel plot

**FigS4.** Forest plot of the leave-one-out analysis results

| 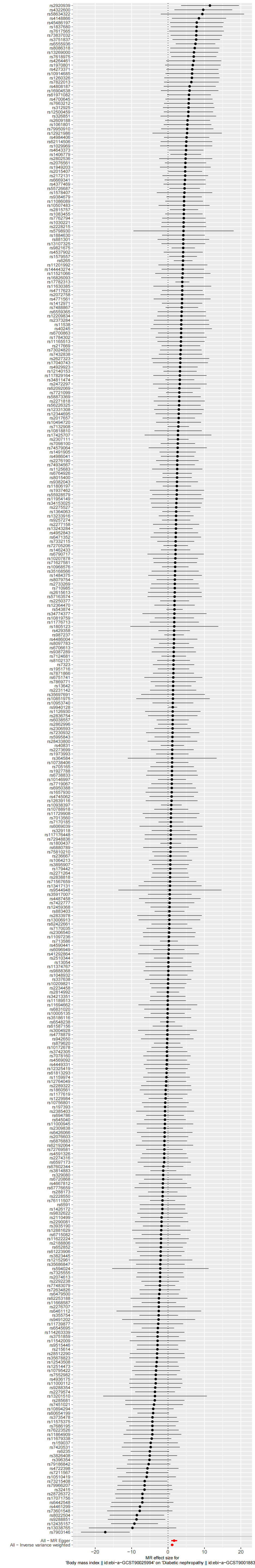 |
| --- |
| **FigS1.** SNPs forest plot |
| 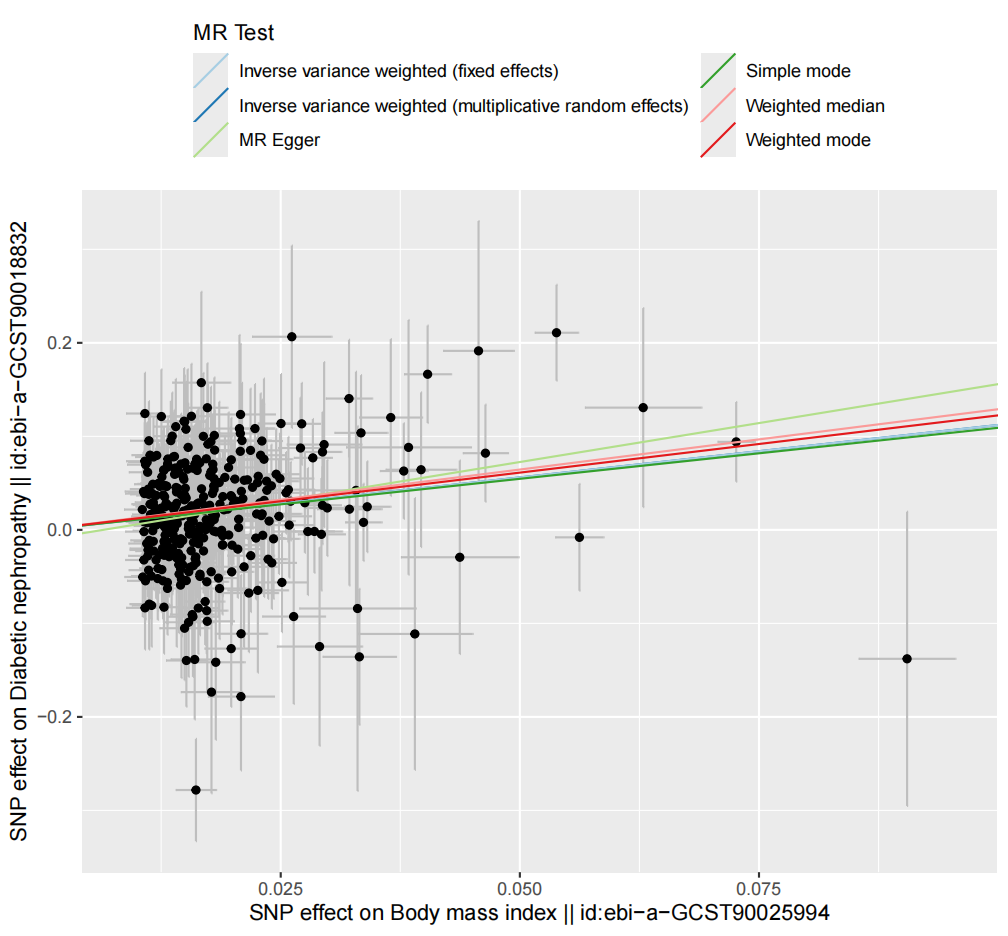 |
| **FigS2.** Scatter plot |
| 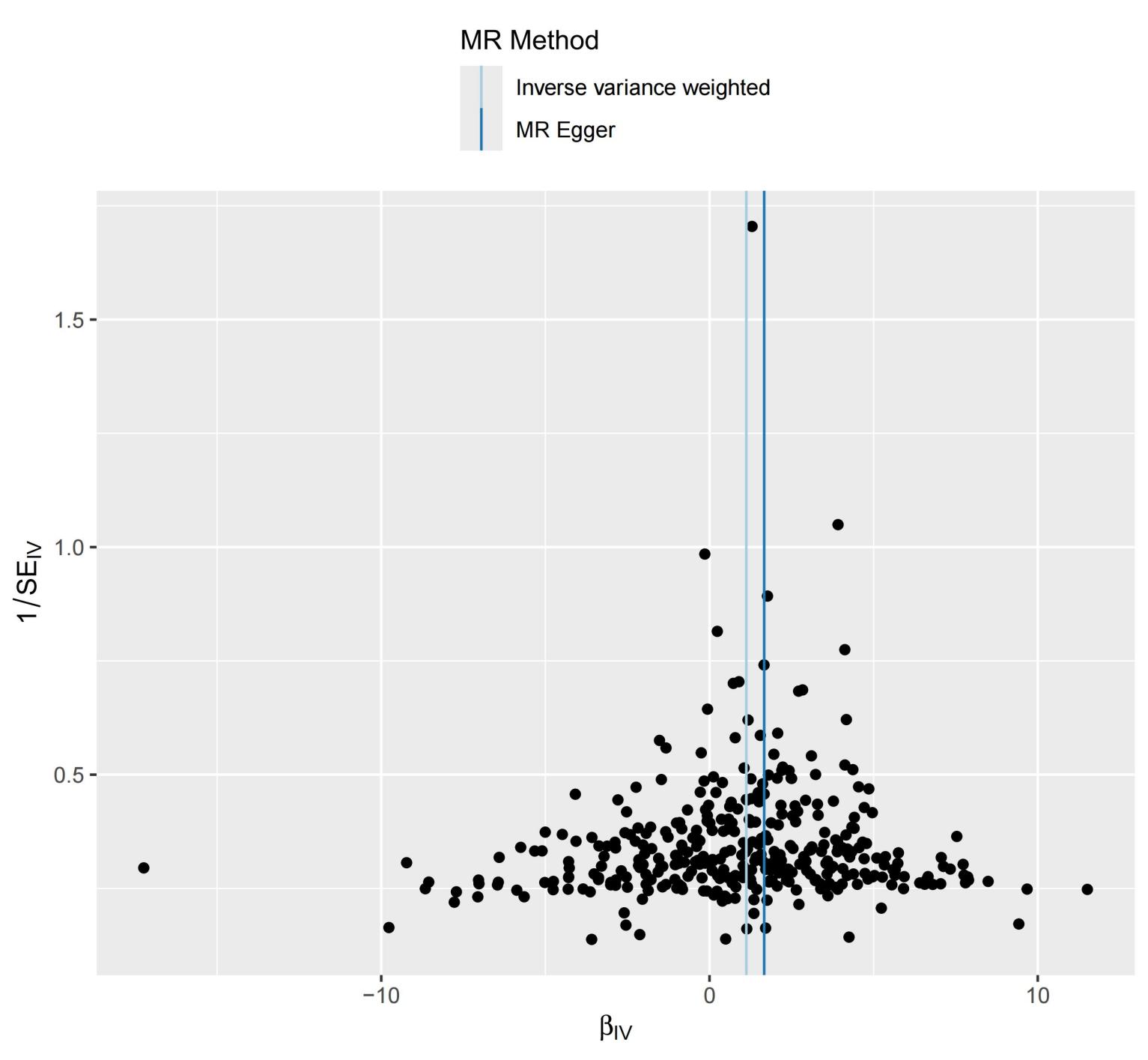 |
| **FigS3.** Funnel plot |
| 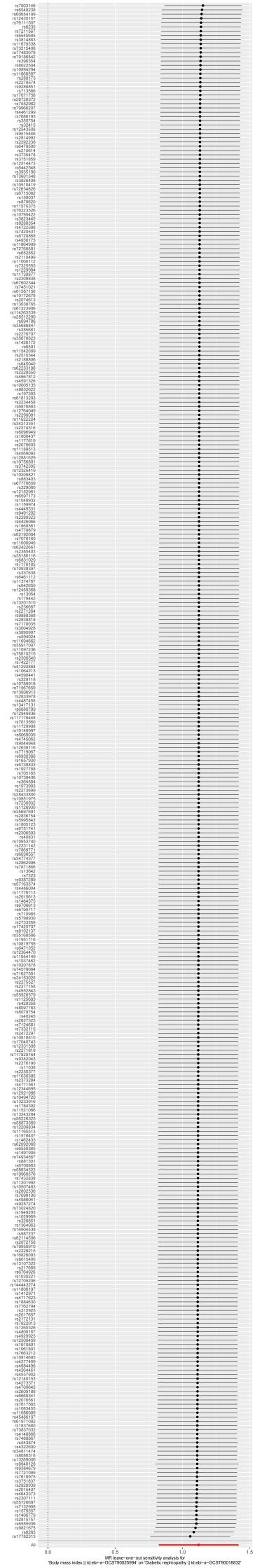 |
| **FigS4.** Forest plot of the leave-one-out analysis results |
